# What works in interventions targeting loneliness: a systematic review of intervention characteristics

**DOI:** 10.1186/s12889-023-17097-2

**Published:** 2023-11-09

**Authors:** N. Morrish, S. Choudhury, A. Medina-Lara

**Affiliations:** Public Health Economics Group, Department of Public Health and Sport Sciences, Faculty of Health and Life Sciences, University of Exeter, South Cloisters, St Luke’s Campus, Magdalen Road, Exeter, EX1 2LU UK

**Keywords:** Loneliness, Effective intervention, Intervention development, Systematic review

## Abstract

**Background:**

Loneliness has been linked to negative health and economic outcomes across the life course. Health effects span both physical and mental health outcomes, including negative health behaviours, lower well-being, and increased mortality. Loneliness is however preventable with effective intervention. This systematic review aims to identify what has worked in interventions for loneliness to guide the development of future interventions.

**Methods:**

Eight electronic databases (Medline, Embase, PsycINFO, Social Policy and Practice, Social Sciences Citation Index, Epistemonikos, CINAHL, Cochrane Library) were systematically searched from inception to February 2022 using terms for intervention and loneliness to identify relevant interventions in the general population. No restrictions on age, socio-economic status, or geographic location were imposed. Studies were to measure loneliness as the primary outcome through a validated scale or single-item question. Case studies were excluded. Additional studies were identified through citation chasing. Extracted data included study and intervention characteristics, and intervention effectiveness for cross-study comparison. Critical appraisal was conducted using the Joanna Briggs Institute and Critical Appraisal Skills Programme tools before the studies were summarised in a narrative synthesis.

**Results:**

Searches identified 4,734 hits, from which 22 studies were included in this review. Of these studies, 14 were effective in reducing loneliness. Additionally, five studies presented unclear findings, and three concluded no decrease in loneliness. Interventions varied between group vs. individual format, online vs. in person delivery, and regarding both intervention duration and individual session length. Furthermore, this review highlighted five key areas when considering designing an intervention for loneliness: use of between session interaction, inclusion of clear learning mechanisms, role of active participation, number of opportunities for group or facilitator interaction, and variation in teaching and learning styles.

**Conclusions:**

Group sessions seem preferred to individual formats, and interaction through active participation and group or facilitator contact appear beneficial, however studies also recognised the importance of a person-tailored approach to delivery. Studies suggest there is no ‘quick fix’ to loneliness, but that learnt practices, behaviours, and community connection should be built into one’s lifestyle to achieve sustained intervention effectiveness. Future interventions should consider longer follow-up periods, male and populations with lower educational levels.

**Supplementary Information:**

The online version contains supplementary material available at 10.1186/s12889-023-17097-2.

## Background

Loneliness can be defined as the subjective experience of perceived lack in quantity or quality of social relationship [1]. Loneliness has been linked to a large number of negative health and economic outcomes across the life course [2]. Health effects span to both physical and mental health outcomes, including negative health behaviours, lower perceived well-being, and eventually up to 50% greater likelihood of mortality in individuals experiencing loneliness [3]. Loneliness can lead to overuse of health care services [4] contributing to overstretched resources, increased waiting times, and impacting health and social care budgets [5]. It is estimated that an effective intervention for older adults who experience loneliness could reduce avoidable future healthcare use by 17% [5]. Consequently, interventions for severe loneliness are expected to prevent avoidable older adult care costs of up to £6,000 per person across 10 years [6], or in the public sector more widely up to £12,000 across 15 years [5] through potential overuse of services. Thus, loneliness is seen to be expensive, but also preventable. An effective intervention has the potential to not only improve health outcomes, but also reduce long-term health expenditure. Concern surrounding loneliness is not however restricted to health and the public sector. Recent research has identified further economic impacts extending to educational attainment, unemployment, and earnings [7–9], with loneliness estimated to cost UK employers £2.5 billion per annum [10] evidencing a wider societal impact and need for cross-sector interventions.

While existing evidence on interventions tackling loneliness have increased in recent years and cover a broad variety of population groups and intervention types, there is a disproportionate focus on older people in Western countries [11]. Research is required to understand the type of interventions that could work across different populations and geographical locations. Additionally, a broader understanding of intervention characteristics, and opportunities for combining or adapting interventions that have been successful in combating loneliness for different population groups, would help advance current research [11]. This review seeks to support these research gaps.

Given the focus of this review on assessing the effectiveness and flexibility of interventions to be adapted to different populations it will not be restricted to a specific age group. This review expands recent research evaluating interventions to alleviate loneliness in young people [12] which suggested socio-demographics, intervention characteristics, and study design do not account for between-study variance in younger people. This review will also complement existing studies which sought to assess and summarise the effect of interventions across age groups, sub-populations, and intervention strategies [12–16]. Our synthesis will place greater emphasis on intervention characteristics of both effective and ineffective interventions to identify common strengths and weaknesses in intervention design, rather than compare effectiveness alone. Where possible it will also incorporate evidence from qualitative studies to gain insight into the why and how interventions may be successful. Finally, this research will address the research gap identified by previous authors who highlight the need to assess and design interventions specifically targeting loneliness [12].

Overall, this systematic review aims to identify what has worked in interventions for loneliness. Thus, this review will guide the development of future interventions for loneliness both in the general population and in specific subgroups of individuals.

## Methods

This systematic review was registered prospectively on PROSPERO (CRD42022313246) and followed the PRISMA reporting guidelines [17].

### Identification of studies

Terms for loneliness and interventions were combined to search title and abstract. Loneliness was captured using the search term ‘lonel*’ and the medical subject heading for loneliness. While related, loneliness is considered distinct from social isolation and thus interventions for social isolation, which is a more objective and physical state [5, 18], are not included in this review.

Intervention terms included ‘intervent*’ and the medical subject heading for intervention consistent with a recent review on alleviating loneliness in young people [12]. Additional terms such as ‘effect*’, ‘control*’, ‘evaluation*’, ‘program*’, ‘treat*’ and ‘manage*’ were considered however reduced specificity was expected to outweigh any benefit from increased sensitivity. The databases searched from inception to 28 February 2022 were: MEDLINE(Ovid), Embase(Ovid), PsycINFO(Ovid), Social Policy and Practice(Ovid), Social Sciences Citation Index(Web of Science), Epistemonikos, CINAHL and The Cochrane Library.

Titles and abstracts were independently screened by two reviewers (NM/SC) identifying articles with the potential to meet the inclusion criteria outlined in Table 1. Full texts were retrieved and again independently assessed for eligibility by the same two reviewers. Any disagreements were resolved through discussion between the two reviewers (NM/SC) and with a third reviewer (AML) where required.

**Table 1:** Inclusion and Exclusion criteria

Inclusion criteria	Exclusion criteria
- General population*. - Loneliness is the primary intervention outcome for effectiveness. - Loneliness measured by a validated loneliness scale or self-report single item question. - English language. - Any publication period.	- Specific populations (e.g. immigrants, twins, veterans, widows)†. - Specific occupations (e.g. dentists)†. - Pre-existing conditions (e.g. chronic conditions, physical or mental health) including studies where at least half the population had low or poor health or had long-term or chronic conditions †. - Focus on related concepts such as social isolation or social connectedness. - Methodological papers, commentaries, letters, editorials, reviews, abstracts, protocols, or trial registrations. - Case studies or studies considering only one individual. - Studies evaluating the outcome of the same intervention and sample of respondents.

* Lessons can be learnt from interventions in different age groups and geographic locations, while socio-economic status should not be a limiting factor where future interventions could be provided free of charge. For these reasons, no restrictions on age, socio-economic status or geographic location were imposed for improved generalisability

† Specific populations excluded to improve generalisability and to reduce confounding from participants with specific loneliness triggers or interventions with specific adjustments for comorbidities. Studies were only excluded where explicitly evidencing a population where most (at least half) were affected by a specific health condition considered likely to require adjustments and limit generalisability to the ‘general population’

Reference lists of relevant systematic reviews identified in database searches were screened and grey literature searched for peer-reviewed publications related to conference abstracts, protocols and trial registrations highlighted in the literature searches. Forward and backward citation chasing was conducted on studies identified as included at full text. These citations were screened following the same process outlined for database searches. Any disagreement was resolved by consensus. Screening was conducted using EndNote 20.

### Critical appraisal

Risk of bias was assessed using the Joanna Briggs Institute (JBI) cohort study critical appraisal tool [19] with results presented using traffic light plots. Additionally, Critical Appraisal Skills Programme (CASP) Randomised Controlled Trial (RCT) checklists [20] were used to assess completeness and standard of randomised controlled trials. Studies were evaluated using both checklists, however as base case the JBI was used for cohort studies, and CASP for RCTs as the CASP checklist facilitates more comprehensive consideration of the results and study impact. Quality appraisal conclusions based on the alternative criteria for each study type are available from the authors on request. Any study not meeting the quality criteria for inclusion were excluded.

### Data extraction and analysis

Relevant information was extracted from each of the included studies and placed into a standardised data form. Extracted data included author; year; study population and participant demographics and characteristics; study type; instrument(s) for assessing loneliness; sample size; intervention characteristics; and outcomes. Data were extracted by one reviewer (NM) and checked by a second (SC). Extracted data were tabulated and described in a narrative synthesis using guidance from Popay and colleagues [21], to identify key themes and patterns. The possibility to conduct a meta-analysis was considered, however given the purpose of the review was to understand intervention characteristics, and the occurrence of large heterogeneity in study and intervention design, a narrative synthesis was concluded better suited to the aims of the study [19, 20].

## Results

**Fig. 1:**
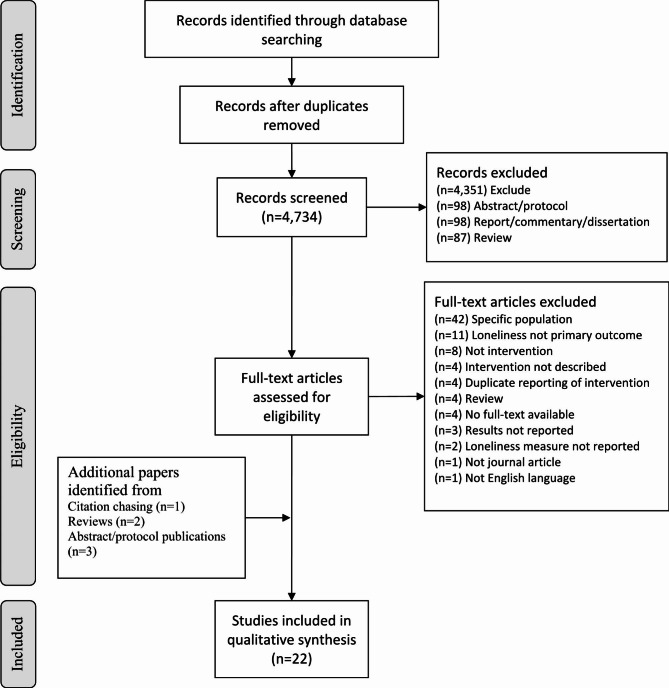
PRISMA diagram Moher, D., Liberati, A., Tetzlaff, J., Altman, D., The PRISMA Group (2009). *Preferred Reporting Items for Systematic Reviews and Meta-Analyses: The PRISMA Statement*. PLoS Medicine, Vol. 6(7). Available from http://journals.plos.org/plosmedicine/article?id=10.1371/journal.pmed.1000097

Details of the study screening process are presented in a PRISMA flow diagram (Fig. 1). Electronic databases yielded 4,734 hits after deduplication. Following title and abstract screening, 100 studies were retrieved at full text from which 16 studies were considered eligible for inclusion in this review. Additional grey literature checks and forward and backward citation chasing brought the total number of included studies to 22 [22–43].

Study population is described in Table 2.

**Table 2:** Study Population

Author year [reference]	Study objectives	Country	Study population				
			Age mean(SD) [range]	Gender (%male)	Ethnicity (%white)	Education	Other characteristics
**Effective**							
Collins 2006 [25]	Evaluate the effectiveness of an educational intervention on older adults’ mastery, loneliness and stress	USA	73.2(8.6) [52–93]	20%	68%	28% did not complete high school; 21% hold degree	70% household income <$19,999 per year.
Creswell 2012 [26]	Evaluate the effect of Mindfulness-Based Stress Reduction program on loneliness	USA	I = 64.4(6.0); WL = 65.2(8.0)	T = 20%; I = 15%; WL = 25%	T = 65%; I = 65%; WL = 65%	Total = 73%; I = 70%; C = 75% college degree /graduate work	T(I,WL): 35%(50,20) retired; 3% (0,5) unemployed; 60%(50,70) employed. Mean(SD): BMI = 25.2(4); MMSE I = 28.0(2), WL = 27.8(2)
Gaggioli 2014 [30]	Evaluate the effects of intergenerational reminiscence on cognitively unimpaired elderly participants focussing on self-esteem, loneliness and isolation, and quality of life	Italy	67.5(6.0)	NR	NR	NR	NR
Larsson 2016 [38]	Evaluate the effects of a social internet-based intervention for older adults vulnerable to loneliness	Sweden	T = 71.2[61–89]; group 1 = 73.4[66–89]; group 2 = 69.0[61–76]	T = 20%; group 1 = 20%; group 2 = 20%	NR	T = 57%; group 1 = 47%; group 2 = 67% university degree	T(group 1,group 2): 30%(33, 27) married/cohabiting; 87%(93,80) participate in offline social activities at least once a week; 70%(67,73) use email at least once a week
Ehlers 2017 [27]	Examine the effect of social support and stress on change in perceived loneliness after an exercise intervention	USA	65.4(4.6)	32%	84%	59% college graduate	59% married. Mean(SD) BMI = 31.0(5.6)
Bouwman 2017 [22]	Investigate whether an online friendship enrichment program can alleviate loneliness	The Netherlands	61.6(7.2) [50–86]	22%	NR	Median level = 8 [1 = primary − 9 = university]	40% have a partner; 74% have children; 72% good health
Cohen-Mansfield 2018 [24]	Understand the efficacy of the I-SOCIAL intervention which addresses social integration barriers for loneliness in old age	Israel	I = 76.6(6.8); C = 79.0(6.6)	I = 21%; C = 17%	NR	I/C = 14 mean years education	I = 15%(C = 14%) married. Mean values I(C): children = 2.2(1.6), MMSE = 27.7(27.9), number medical diagnoses = 2.8(2.6), subjective health [range 1–4] = 2.4(2.2)
Hwang 2019 [32]	Develop an understanding of the experience of living with loneliness and social isolation	Canada	76.6[65–88]	13%	NR	NR	69% live alone; 13% caregivers
Kall 2020 [33]	Investigate the long-term effects of an internet-administered programme based on CBT principles	Sweden	T = 47.2(17.6); I = 45.6(16.7); C = 48.8(18.4)	T = 29%; I = 28%; C = 30%	NR	T = 66%; I = 65%; C = 67% university degree	T(I,C): partner/married 32%(33,30); previous treatment for mental illness 47%(53,41); no use 62%(58,65), previous use 12%(11, 14), ongoing use 26%(31,22) of psychopharmaceutic medication
Ghanbari 2021 [31]	Investigate the effect of a program promoting coping and assessment processes on loneliness in children	Iran	I = 10.6(1.4); C = 10.2(1.3)	NR	NR	100% in school grades 3–6	I(C): 20%(27) only children; 54%(43) oldest child
Fong 2021 [29]	Evaluate the effectiveness of a community-based intervention designed to increase neighbourhood identification and reduce loneliness	Australia	18+	17%	NR	51% university degree	20% from most disadvantaged areas, 43% average SES, 35% most advantaged areas
Kall 2021 [34]	Investigate the efficacy of two internet-based interventions for loneliness based on cognitive behavioural therapy (CBT) and interpersonal psychotherapy (IPT)	Sweden	T = 47.5(16.4); IPT = 49.7(16.4); CBT = 47.2(15.9); WL = 43.9(17.1)	T = 24%; IPT = 16%; CBT = 28%; WL = 32%	NR	T = 69%; IPT = 74%; CBT = 62%; WL = 77% university degree	T(IPT,CBT,WL): 18%(21,22,6) married/cohabit; 65%(68,56,79) live alone; 68%(68,68,71) employed/student; 57%(63,56,44) previous mental health treatment; 46%(53,40,44) previous/current psychotropic medication; 45%(53,36,47) loneliness from an event. Mean(SD): years duration of loneliness IPT = 11(12), CBT = 12(16), WL = 11(12); age lonely onset IPT = 27(22), CBT = 25(19), WL = 27(19).
Nazari 2021 [39]	Determine the impact of a social participation educational program on the feeling of loneliness for the elderly	Iran	Men: I = 46.0(38.7); C = 57(47.5) Women: I = 73 (61.3); C = 63(52.5)	I = 39%; C = 48%	NR	NR	I(C): 81%(83) married; 19%(18) widow; 8%(7) live alone; 53%(41) unemployed, 60%(52) housewife, 5%(7) employed
Kotwal 2021 [36]	Assess the effect of a peer intervention addressing loneliness in low-income older adults	USA	Median = 70.0, IQR[66–76], range[59–96]	58% (66% in qualitative interview)	42% (33% in qualitative interview)	12% college graduate or more	14% married/partner; 88% live alone; 18% LGBT; 62% English primary language; 11% veterans; 36% at least 1 functional impairment; 36% depression; 66% high loneliness.
**Can’t tell if effective**							
Steven 2000 [43]	Evaluate an educational friendship program for older women to alleviate loneliness	The Netherlands	I = 63.4; C = 69.8	0%	NR	NR	I = 25% married; 38% widowed; average 3 children (C = 2 children). Total = 75% live alone
Rolandi 2020 [40]	Investigate the long-lasting effect of Social Network Site use training in oldest-old adults on loneliness in the context of COVID-19 quarantine	Italy	T = 81.8(1.4); trained= 82.0(1.6); untrained = 81.6(1.2)	T = 48%; trained = 47%; untrained = 49%	NR	Mean(SD) education: T = 9(3); trained = 9(3); untrained = 9(4)	T(trained, untrained): 37%(38,36) live alone. T(trained, untrained) mean ± SD GDS score = 1.9 ± 1.9 (2.0 ± 1.9, 1.8 ± 2.0); MMSE score = 28.4 ± 1.5 (28.3 ± 1.4, 28.4 ± 1.5)
Caputi 2021 [23]	Assess the effects of theory of mind training on loneliness	Italy	9.7(0.9) [9, 10]	NR	NR	NR	Median socioeconomic status = 6[range 2–9]
Kanter 2021 [35]	Investigate whether a brief mobile based intervention for social relationships can decrease loneliness and improve relationship quality during the COVID-19 pandemic	USA	T = 41.7(15.0); I = 41.2(15.1); C = 42.3(14.9)	T = 18%; I = 20%; C = 17%	T = 77%; I = 76%; C = 78%	NR	T(I,C): 44%(43, 46) married; 51%(54,49) no children; never diagnosed with major depressive disorder 72%(73,71), OCD 94%(94, 94), generalised anxiety disorder 68%(70,66), social anxiety disorder; 89%(90,88)
Shapira 2021 [42]	Explore the effects of a short-term digital group intervention aimed at providing cognitive behavioural and mindfulness tools and skills to reduce loneliness	Israel	T = 72[65–90]; I = 72.1(5.3); C = 71.7(6.8)	T = 20%; I = 19%; C = 22%	NR	T = 71%; I = 76%; C = 59% tertiary education	T(I,C): 37%(38, 35) live alone
**Not effective**							
Fields 2021 [28]	Evaluate the effect of a community-based digital intervention on loneliness, perceived social support, and technology use in isolated older adults	USA	T = 75.0(7.9); I = 74.0(8.5); WL = 76.0(7.4)	T = 47%; I = 52%; WL = 43%	T = 60%; I = 67%; WL = 53%	T = 45%; I = 50%; WL = 40% completed high school or less	T(I,WL): 69%(77,62) household income <$20,000 per year; 13%(12,13) limited English proficiency; 32%(33,30) no cell phone; 54%(56, 53) fair or poor mental health; 21%(29,14) frequent mental distress; 45%(46,44) frequent physical distress; 35%(41,31) frequent functioning interference
Sandu 2021 [41]	Explore the impact of a Good Neighbour Program on reducing loneliness in older adults during the COVID-19 pandemic	USA	60+	38%	67%	NR	60% live alone; 67% low-income; 3% veterans.
Kramer 2022 [37]	Identify whether Embodied Conversational Agents (ECA) could decrease loneliness in older adults	The Netherlands	73(5.33) [65–85]	44%	NR	59% completed college or university	Mean(SD)[range]: eHealth literacy score 29.3(4.4) [15–34]; malnutrition risk 9.7(1.4) [7–11].

T = total; I = intervention; C = control, WL = waitlist

CBT = cognitive behavioural therapy; IPT = interpersonal psychotherapy

MMSE = mini mental state examination; BMI = body mass index; GDS = geriatric depression scale

Most studies were conducted in high-income Western countries (20/22), largely based in the USA (n = 7), with the remaining two studies conducted in Iran (n = 2). Studies were predominantly conducted on older adults aged over 60 (n = 15), though also covered the adult population with mean age in 40s (n = 5), and only two were conducted with children (n = 2). Most studies (n = 18) included predominantly female participants, including one study conducted only on women. Ethnicity was reported in seven studies, six of which were majority white populations while one had majority non-white ethnicity [36]. Education was reported in 15 studies; where reported, completion of college or university was achieved between 12% [36] and 73% [26]. Sample size ranged from 30 to 1,420 participants. Among the loneliness measures used, the UCLA loneliness scale [44] was the most popular (n = 15), however this tool was used in a variety of its different modalities (e.g. 20-item vs. 4-item). A further 5 loneliness scales, and single-item questions, were used across the included studies, either alone or in combination with each other. Newspaper advertisement was the most reported method of recruitment (n = 9).

Session format is a key intervention characteristic as detailed in Table 3. Included studies were largely published from 2020 onwards (n = 14). Of those published before 2020 only one intervention was conducted online [22]. In total, loneliness interventions were delivered in person (n = 12), online (n = 7), as a hybrid online/in person combination (n = 2), or by phone (n = 1). Half of the studies were randomised, crossover, or interventional trials (n = 11). Remaining studies used non-randomised study designs such as pre-test post-test (n = 3) and repeated measures/follow-up (n = 2). Further details regarding study design and data collection can be found in Appendix 2.

**Table 3:** Session format

Author year [reference]	Intervention duration	Total number of sessions	Duration of each session	Group / individual	Online / in person
**Effective**					
Collins 2006 [25]	16-weeks	16 classes	2 h	Group	In person
Creswell 2012 [26]	8-weeks	8 sessions + 1 retreat	120-minute sessions. Day-long retreat	Group	In person
Gaggioli 2014 [30]	3-weeks	3 sessions	2 h	Group	In person
Larsson 2016 [38]	34-weeks	Between 2–5 group and 1–16 individual meetings	Maximum 1.5 h	Individual and/or group	In person and/or online
Ehlers 2017 [27]	24-weeks	72 sessions	1-hour	Group	In person
Bouwman 2017 [22]	6-weeks	NR	NR	Individual	Online
Cohen-Mansfield 2018 [24]	NR	Mean 4 [range 0–7] group sessions, mean 5 [range 1–13] individual meetings	NR	Individual and/or group	In person
Hwang 2019 [32]	12-weeks	24 sessions	2 h 35 min	Group	In person
Kall 2020 [33]	8-weeks	8 modules	NR	Individual	Online
Ghanbari 2021 [31]	4-weeks	16 sessions	30 min	NR	In person
Fong 2021 [29]	1 day	1 event	NR	Individual and/or group	In person and/or online
Kall 2021* [34]	9-weeks	9 modules	NR	Individual	Online
Nazari 2021 [39]	5-weeks	5 sessions	60–80 min	Group	In person
Kotwal 2021 [36]	Between 6- and 24-months	NR	NR	Individual and/or group	In person
**Can’t tell if effective**					
Steven 2000 [43]	NR	12 lessons	NR	Group	In person
Rolandi 2020 [40]	5.5-weeks	5 sessions + 6 tutoring	2 hours^φ^	Group	In person
Caputi 2021 [23]	5-weeks	5 sessions	50-minutes	Group	In person
Kanter 2021 [35]	16 days	14 text messages	Between 5 and 12 min	Individual	Online, mobile based
Shapira 2021 [42]	3.5-weeks	7 sessions	1-1.5 h	Group	Online
**Not effective**					
Fields 2021 [28]	8-weeks	8 sessions	NR	Individual	Online
Sandu 2021 [41]	1 year	NR	Median 11-minute phone call	Individual	Over the phone
Kramer 2022 [37]	NR	NR	NR	Individual	Online

Φ Casanova 2021

The most common control method was a waitlist control group with participants receiving the intervention later after an initial control period, providing a more ethical service (n = 8). The ethical challenge of denying loneliness intervention was overcome in other studies by the provision of different interventions (n = 3), provision of educational materials (n = 1), and drawing from a representative sample of a national longitudinal survey (n = 1). Otherwise, no support was provided to the control group (n = 2), no control group was included (n = 2), or the control group was not clearly reported (n = 5).

### Effective interventions

A total of 13 studies were effective achieving a sustained reduction in loneliness levels with statistical significance (p < 0.05, or as stated by the authors). As detailed in Table 2, these studies aimed to alleviate loneliness through interventions based primarily on: social connection, friendship or community integration such as community exercise programs and neighbour days (n = 6), education (n = 1), mindfulness-based stress reduction (n = 1), intergenerational reminiscence (n = 1), exercise (n = 1), understanding loneliness (n = 1), coping strategies (n = 1), and cognitive behavioural therapy (n = 1). The study utilising cognitive behavioural therapy (CBT) principles [33] showed a significant reduction in loneliness for the entire sample, both intervention and waitlist control. In this case, at follow-up the intervention had been received by both groups, providing access to the same material and opportunity for facilitator contact. An additional study focussed on both CBT and interpersonal psychotherapy (IPT) and found only CBT to yield a statistically significant reduction in loneliness when compared to a waitlist control group [34]. Given the study revealed a statistically significant intervention (CBT) it is considered ‘effective’ for the purpose of this review, bringing the total number of effective interventions identified to 14.

A summary of intervention effectiveness is presented in Table 4 with further detail of quantitative loneliness outcomes and key study conclusions provided in Appendix 3. Studies are grouped and described according to their effectiveness. Of the 14 effective interventions, 79% (11/14) had some form of in person interaction. Group sessions accounted for 71% (10/14) of effective interventions, of which four studies also included a concurrent opportunity for individual sessions. Otherwise, interventions were delivered individually (n = 3), or setting was not reported (n = 1). Included sessions lasted for at least 30-minutes and the full intervention ranged from a duration of one-day [29] to two-years [36] while the majority of effective interventions occurred for fewer than six-months (n = 11, 79%). Study duration of between nine- and 34-weeks yielded only effective interventions (n = 5).

**Table 4:** Intervention Effectiveness

Author year [reference]	Effective	Noted differences across groups / characteristics
**Effective**		
Collins 2006 [25]	**Yes** – reduction in mean loneliness with statistically significant improvement	Greatest reduction of loneliness observed among **ethnic minorities**, amongst whom **lowest income** reported significantly less loneliness than highest income. Minority participants with **highest education** had significantly greater improvement compared to second and third levels but were not significantly different from those at lowest education level
Creswell 2012 [26]	**Yes** – reduction in mean loneliness and statistically significant model interaction	**More participants dropped out of intervention group**; difference was marginally significant. No significant differences between groups regarding baseline demographics or pairwise comparison. No significant differences between dropouts in primary treatment trial and treatment completers
Gaggioli 2014 [30]	**Yes** – reduction in mean loneliness with statistical significance for general and emotional	The decrease in emotional but not social loneliness could be due to the **nature of the intervention**, in particular reminiscing to promote feelings of togetherness and intimacy
Larsson 2016 [38]	**Yes** – statistically significant reduction in mean loneliness for both intervention-control sequences, and negative percentage change	No significant difference between T3 and T2 for group 1 [I/C]. Majority of participants were women living alone, representing those more vulnerable to loneliness. However, despite this study population characteristic, none of the participants reported high baseline loneliness, indicating the intervention was tested on a group with low to moderate loneliness. Interaction between intervention and sequence did not achieve statistical significance
Ehlers 2017 [27]	**Yes** – reduction in mean loneliness and statistically significant latent change score	Persons with **higher levels of baseline loneliness** at demonstrated greater decreases over the course of the intervention. Mediation model found **greater decrease in stress** explained greater reductions in loneliness, and **increased social support** was directly related to decreased loneliness. Collectively changes in social support and stress explained around 26% of the variability in change in loneliness. **Mode of exercise intervention did not** account for individual differences in loneliness with similar change in loneliness observed across different intervention conditions
Bouwman 2017 [22]	**Yes** – reduction in mean loneliness and statistically significant linear regression coefficients	**Number of lessons** did not affect loneliness in the full group, and only slightly increased loneliness in the light program. **Regulative coping** is more effective than active coping in alleviating today’s loneliness. Higher levels of loneliness when the **assignment did not go well** (full program). **Practicing** (e.g. assignments) is more effective than just reading about coping strategies
Cohen-Mansfield 2018 [24]	**Yes** – reduction in mean loneliness, significant decline in loneliness for intervention as compared to control group	Provided option of group or individual based on pilot work which found **some people were not comfortable in groups**, or not initially willing to participate in groups. Individual sessions allow work on specific barriers and solutions, group sessions allow participants to practice and share solutions. Significant effect for interaction of intervention group by **time**. **Baseline loneliness** and **number of group sessions** attended are significant predictors of the final loneliness score. Impact of group setting likely reflects both the impact of the group and that **those who attended group sessions were more ready** to enhance social activities and tackle loneliness
Hwang 2019 [32]	**Yes** – qualitative and quantitative decreases in loneliness stated	UCLA loneliness score showed significant decrease in loneliness and de Jong Gierveld showed significant decrease in emotional loneliness. No significant change on Lubben score, possibly due to the **short intervention period**
Kall 2020† [33]	**Yes** – reduction in mean loneliness, positive effect size, and statistically significant model outputs for intervention and waitlist	Nonsignificant relationship between post-treatment loneliness and ‘dose’ (i.e., loneliness not significantly related to **number of completed modules** or **average treatment time** from therapist)†
Ghanbari 2021 [31]	**Yes** – significant reduction in mean loneliness, and difference between intervention and control	NR
Fong 2021 [29]	**Yes** –statistically significant model outputs for reduced loneliness	Greater baseline loneliness was reflected in greater loneliness at follow-up. The higher the **level of education** the larger the reduction in loneliness at follow-up
Kall 2021* [34]	**Yes** – reduction in mean loneliness, CBT favoured over waitlist with statistical significance. **No** - IPT did not present statistically significant results	Found significant heterogeneity in the initial level of loneliness and slope
Nazari 2021 [39]	**Yes** - reduction in mean loneliness with statistically significant difference between intervention and control after the study	No statistically significant difference across gender, while other studies have shown that women are lonelier than men
Kotwal 2021 [36]	**Yes** – statistically significant reduction in loneliness score	Suggests the one-to-one intervention was more successful than previous interventions using telephone-based support, gatekeepers or clinical case managers. Suggest this could be due to the **social experience** rather than treatment with medical providers; **flexibility** of number, frequency and goal of sessions; motivational interviewing and companionship to promote safety; focus on **unique needs of participants**
**Can’t tell if effective**		
Steven 2000 [43]	**Can’t tell** - reduction in mean loneliness for both intervention and control groups, greater mean change score for intervention than control	NR
Rolandi 2020 [40]	**Can’t tell** – non-significant differences for total loneliness score, some benefit to specific feelings	Between-group differences observed for individual UCLA scale items e.g. cross-sectional analysis for feeling left out highlighting **potential benefit of social network site use for specific loneliness feelings**
Caputi 2021 [23]	**Can’t tell** – reduction in mean loneliness and statistically significant model outputs in short-term but not long-term	Significant negative effect of **verbal ability**. Reduced loneliness among ToM training group likely due to engagement in discussions about **different perspectives** while no-ToM group discussed non-social stories. Higher vocabulary scores predicted lower loneliness thus **language has a protective role** against high perceived loneliness
Kanter 2021 [35]	**Can’t tell** – reduction in mean loneliness, statistically significant intervention effect at start but lost by final day	The effect of the intervention on loneliness increased over the intervention period and was **strongest on the last day** of the intervention, **reducing after the intervention was discontinued**. No covariates were significantly associated with differing intervention effects. Characteristics associated to higher odds of missing or incomplete surveys include: assigned to intervention, being farther along in the study, younger age, living farther north. Low participating participants more likely: younger, non-white, unmarried, income <$10,000 per year
Shapira 2021 [42]	**Can’t tell** – reduction in mean loneliness but only achieved statistically significant difference at T1	Significant main effect of time-by-group interaction indicating groups differed in loneliness post-intervention. Main effect of time did not reach statistical significance. Lack of continued decrease after 1-month follow up (T2) implies group contacts can lead to a decline in loneliness once interactions become less **frequent**.
**Not effective**		
Kramer 2022 [37]	**No** – no decrease in loneliness	None of the demographic characteristics were significantly associated with loneliness. **Number of chat messages** correlated with, but did not predict, loneliness
Fields 2021 [28]	**No** – overall no quantitative change in loneliness	Authors suggest the lack of quantitative change could be due to additional pre-existing contextual factors in the daily lives of participants (e.g. physical disability, lack of close friends or living relatives etc.) making loneliness more systemic and harder to change. Additionally, could be confounding from tackling the digital divide alongside loneliness
Sandu 2021 [41]	**No**	No significant relationship between change in loneliness and either duration or number of calls. However, did notice a trend towards significance in relationship between UCLA loneliness score and increasing duration of calls

CBT = cognitive behavioural therapy; IPT = interpersonal psychotherapy; ToM = Theory of Mind

† Kall 2020 showed a significant reduction in loneliness for the entire sample (intervention and control). Included as effective given the intervention (resource access and facilitator contact) had been received by both groups (intervention and waitlist control) by the time of follow-up loneliness measurement

* Kall 2021 included an effective CBT intervention, though also included an ineffective IPT intervention

Studies were grouped into five key areas for consideration regarding study design. These groups were identified based on themes arising from the included papers. The first area identified was the *use of between session interaction* which was included in six studies through: practice (n = 3), facilitator contact (n = 4), and/or group contact with other participants (n = 2). The second area considered the role of *clear learning mechanisms* which were present in 11 studies and covered opportunities to learn about: behavioural change techniques (n = 7), friendship or community connection (n = 5), and/or health education (n = 2). Third a role of *active participation* was recognised. Some form of active participation was observed in 10 of the included effective interventions arising through: mindfulness exercises (n = 3), physical exercise (n = 3), community or social events (n = 4), and/or specific assignments (n = 3). Fourth identified were a number of *opportunities for group and/or facilitator interaction*. Eight studies incorporated at least one group or facilitator interaction opportunity. These included: memory/shared experience (n = 2), group discussion (n = 5), group session practice (n = 2), facilitator feedback (n = 3), and online or phone messaging (n = 3). Finally, the fifth area of note were *teaching and learning styles* identified across eight effective interventions. These included: reading text (n = 3), visual guides or images (n = 3), pre-recorded video or audio (n = 1), interactive teaching (n = 3), imagined scenarios or role play (n = 3), and/or answering questions (n = 3).

Several differences in effectiveness are also reflected in the studies themselves as a number of authors reported possible reasons for noted differences across groups in either loneliness levels or intervention effectiveness. These details are recorded in Table 4. Factors included population demographics (e.g. ethnicity, income, education, verbal ability, baseline loneliness), and the nature or characteristics of the intervention (social vs. emotional focus, number of lessons, perceptions of assignments going well, practice, short intervention period, one-to-one meetings, social experience, flexibility, companionship, focus on unique needs, voicing different perspectives, intervention discontinuation, duration of calls).

### Interventions of unclear effectiveness

Five studies were unclear in whether the intervention yielded a statistically significant reduction in loneliness. This lack of clarity most often arose from reports that short-term effects were lost in the long-term [23, 35, 42]. A further study by Rolandi [40] failed to achieve statistical significance in their findings for the overall loneliness score requiring caution in their interpretation. This study did however find a statistically significant difference to specific items of the UCLA loneliness scale, in particular feeling ‘left out’, and so is not considered fully ‘ineffective’ for this review. The final study provided no baseline estimate for loneliness and identified a significant reduction in loneliness for both the intervention and control group alongside some evidence of increased loneliness in the intervention [43]. The main aims of these interventions were to alleviate loneliness through promoting friendship and social relationships (n = 2), online social network use (n = 1), theory of mind training (n = 1), and CBT and mindfulness (n = 1). Interventions were split across online (n = 2) and in person (n = 3) formats, and between group (n = 4) and individual (n = 1) settings. Group sessions occurred over no more than a six-week period and lasted between 50- and 120-minutes. The individual format was conducted via text message with tasks lasting between five- and 12-minutes.

Interventions included the full spectrum of *between session interaction* techniques identified in effective studies. Regarding the role of *learning mechanisms*, compared to effective interventions, interventions where effectiveness could not be clearly deduced did not provide health education, however included an additional mechanism, learning about the internet or smartphones (n = 1). Only two studies with unclear effectiveness utilised methods of *active participation* [35, 42], though these did not include any community/social events. All categories reflecting opportunities for *group and/or facilitator interaction* were represented across interventions where effectiveness was unclear with three studies including multiple forms. Finally, as observed in effective interventions, interventions of unclear effectiveness utilised a wide range of *teaching and learning styles* with evidence of all but visual guides or images in the included studies.

### Ineffective interventions

Three studies concluded there to be no decrease in loneliness as a result of the intervention, thus were considered ineffective [28, 37, 41]. These interventions aimed to reduce loneliness through social support and technology use (n = 1), a Good Neighbour Program (n = 1), and introducing Embodied Conversational Agents (computer generated humans) (n = 1). These ineffective interventions were delivered online (n = 2) or by phone (n = 1). All interventions deemed not to be effective were delivered using individual one-to-one session format. Not enough data were available to identify trends in intervention duration or number of sessions. Only one study had data on individual session duration being an 11-minute phone call [41], one of the shortest across all interventions included in this review.

Of the three ineffective interventions, only Fields reported any *between session interaction* or practice which was incorporated through activity booklets [28]. No ineffective intervention sought to achieve friendship or community connection as a *learning mechanism*, otherwise, all aforementioned learning mechanisms were observed in ineffective interventions, again including learning about the internet or smartphones (n = 1). Only one ineffective intervention [28] included an element of *active participation* being through specific assignments. Only one characteristic related to *group or facilitator interaction* was present in ineffective interventions, being chat or messaging online or by phone (n = 2). Finally, regarding *teaching and learning style*, ineffective interventions were more limited than other interventions in their range, with no evidence of engagement through video or audio, interactive teaching, or role play.

Additionally, one study [34] considered both CBT and IPT intervention compared to a waitlist control group. While CBT was effective, IPT was not effective. Again, this intervention was delivered individually, online through text and images, with between session interaction through messaging the facilitator/therapist online.

**Fig. 2:**
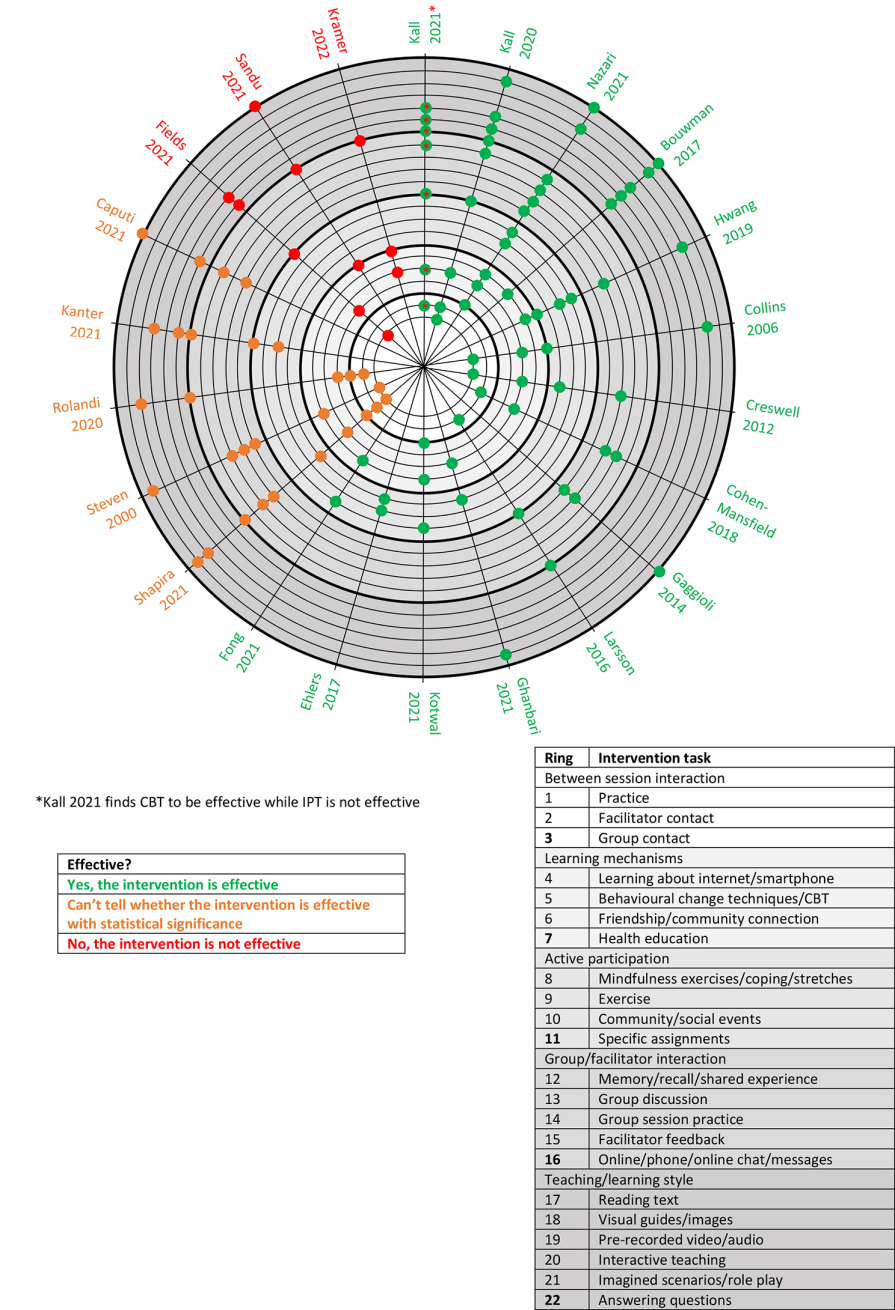
Intervention structure, aims, and tasks

While the focus of each study has been detailed above, a wide range of sub-characteristics and intervention design details are also reported in Table 3; Fig. 2, with additional detail available in Appendix 4.

### Quality appraisal

No study fully met all critical appraisal criteria using the JBI cohort or CASP RCT study checklists. The majority of studies were of low-moderate quality (40–60% of the criteria met). Areas of limitation in each study were not considered critical enough to exclude any study based on the assessment. Further detail of quality appraisal using the JBI cohort and CASP RCT can be found in Appendix 1, and the JBI RCT and CASP cohort checklists are available on request.

Using the JBI critical appraisal tool for cohorts (n = 11) confirmed that, where applicable, most studies recruited from the same population, measured exposures and outcomes in a valid and reliable way, and used appropriate statistical analysis. No study included participants free of the outcome at the start which was to be expected as the aim was to measure a reduction in loneliness. Limitations were also observed in the identification of confounding factors and incomplete follow-up, alongside strategies to deal with both confounders and follow-up.

A sub-group of studies were also evaluated using the CASP checklist for RCTs (n = 11) which supplied additional quality criteria. The CASP RCT checklist found that all studies addressed a clearly focussed issue, randomised intervention assignment, had similar study groups at baseline, and delivered the same level of care to all study groups. The checklist also highlighted that no study blinded participants, while the majority also neither blinded investigators nor people assessing/analysing the outcome. Thus, the basic study design was concluded valid for an RCT for all included studies.

## Discussion

This review summarises recent evidence on loneliness interventions with over 60% of included papers published from 2020 onwards. Intervention characteristics are considered and broken down to understand what works in interventions for loneliness. Noted differences across population groups, and various session formats, such as groups vs. individual or in person vs. online, are first discussed. This review then considers the themes, structures, and tasks used to form the intervention content including between session interaction, learning mechanisms, active participation, group and facilitator interaction, and teaching and learning style. Together these discussion points form overarching insight into what works (effective intervention), and what perhaps does not (ineffective) for different groups.

Consistent with other reviews [8], the UCLA loneliness scale proved most popular, though used in various modalities. It is clear from the wide variety of loneliness measures used that there is no standard measure of loneliness used in research. The use of one such measure would greatly enhance the ability to measure intervention effectiveness, and compare across interventions, more effectively. Studies frequently compared their intervention group to a waitlist control who received the intervention resources at a later date, providing a more ethical comparator as support was still provided in the longer term. Recruitment was most commonly conducted through newspaper advertisements, perhaps reflecting the older participant demographics and inclusion of in person studies. Most studies were conducted in the adult population, or more specifically individuals aged 60 and over. There were also two interventions designed for children, one proving effective [31] and the other with unclear effectiveness [23] suggesting more research is needed targeting this specific population. Furthermore, no studies were found to focus on young adult populations. While age and gender were generally well reported, detail on ethnicity was more sparse. Collins [25] found greatest reduction in loneliness amongst ethnic minorities, while existing research by Salway [47] has previously highlighted higher risks of loneliness amongst ethnic minority groups [47]. However in this current review, most included studies represented majority white participants indicating a key area for improvement in loneliness interventions [47].

While one included study proved effective in a sample of lower education level and higher proportion of men [36], effective interventions were generally conducted in majority female and higher education populations [48, 39]. Furthermore, existing UK national reports [49] have shown that women are lonelier than men, or at least report greater levels of loneliness [49]. Overall however included studies do not provide a conclusive response on the impact of gender in intervention effectiveness, given studies such as Nazari [39] found no statistically significant difference across gender. Thus, research should consider whether different interventions targeting loneliness may be more appealing, and so more effective across different genders.

A large proportion of studies concerned individuals of higher education levels. Individuals with highest education levels had significantly greater improvement in loneliness [25, 29]. Furthermore, Caputi [23] suggested higher vocabulary scores predicted lower loneliness and thus has a protective role against high perceived loneliness. There is however also a need to focus on interventions which are more accessible to individuals with lower education levels. This is particularly important when thinking about transferability to lower income or less developed countries. Furthermore, this relationship of loneliness to education and language is of particular importance given the previously observed association between loneliness and both academic attainment and socio-economic status [11].

Hwang [32] suggested a short intervention period could limit findings of significant change in loneliness. The most common reason an intervention was unclear on whether it was effective or not arose from lack of sustained impact, with intervention effects being lost in the long-term [23, 35, 42]. One such study [35] reported the effect of the intervention was strongest on the last day of the intervention, reducing after the intervention was discontinued [35]. This suggests that once interventions cease, their improvements can revert. Thus, loneliness interventions need to be built into lifestyles, not ‘quick fixes’. Not all ‘effective’ interventions were evaluated over such a long follow-up period with most taking final measures at the end of the interventions, or shortly after (within one-month). There is therefore potential for other interventions initially considered to be effective, to have no long-term effect. More work is needed to resolve the lack of clarity arising from the ‘unclear effectiveness’ categorisation regarding follow-up measures and intervention duration to establish whether interventions that seem effective over a short period, with loneliness measured in the final session, remain so at a later date. This review therefore suggests interventions targeting loneliness should be evaluated with a longer follow up period, continuing after the conclusion of the intervention, to confirm longevity in reduction of reported loneliness.

Findings suggest an intervention of at least two-months to be optimal as the effectiveness of some interventions was unclear before that point. One paper [29] suggested an intervention lasting only one day could be effective [29], however did not measure lasting community building outcomes, alongside other potential study limitations arising from the less controlled study environment. Additionally, an outlier also occurred in an intervention of 52 weeks, conducted by Sandu [41], which proved ineffective. In this case it is possible that effectiveness was more dominantly impacted by other outlying factors such as over the phone delivery, or short session duration, as no effective intervention was observed with sessions under 30-minutes long in this review. Additionally, Bouwman [22] found number of lessons had little effect on loneliness, while Kall 2020 [33] found loneliness was not significantly related to number of completed modules or average treatment time, and Kramer [37] who concluded the number of chat messages correlated with, but did not predict, loneliness. Meanwhile, Cohen-Mansfield [24] found that the number of group sessions attended was a significant predictor of final loneliness score. This could, however, reflect both the impact of the group and that those who attended group sessions were more ready to enhance social activities and tackle loneliness, as noted by the authors, rather than simply the number of sessions.

Group sessions appeared preferred with all ineffective interventions delivered on an individual basis. Studies revealed mixed results for online interventions, while conversely in person interventions were predominantly effective. It was also noticeable that online interventions dominated from 2020 onwards, a likely side-effect of the COVID-19 pandemic. While this was a necessary adaptation because of the pandemic it is clear the success of online interventions is not universal. Overall, most included studies were delivered in an individual-online or group-in person format with little exploration of online group interventions or individual in-person. Cohen-Mansfield [24] provided the option of group or individual based on pilot work which found some people were not comfortable in groups, or not initially willing to participate in groups suggesting some benefit to a person-tailored research or availability of multiple format options. Benefits to focussing on the unique needs of participants were also concluded by Kotwal [36]. Future research could explore the benefits of alternative combinations in order to better understand the more important elements of intervention format and delivery. It should however be noted that for online and individual sessions such delivered as a result of the COVID-19 pandemic, there is potential for confounding from the isolating effects of COVID-19 and subsequent lockdowns. Specific care should now be taken to understand the consistency of intervention preferences in a more online post-COVID world.

Intervention structure and tasks were seen to span five key areas including between session interaction, learning mechanisms, active participation, group or facilitator interaction, and teaching or learning style. It was clear that interactive elements were more popular in effective interventions through group or facilitator interaction, and through active participation. Both elements displayed limited use in studies of unclear effectiveness. They were also notably missing from ineffective interventions with only one category of group or facilitator interaction, being online/phone chat or messaging [37, 41], and only one case of active participation through specific assignments [28]. Consistent with these findings, Bouwman [22], who presented an effective intervention, found practicing, through for example assignments, to be more effective than just reading about coping strategies. Ineffective interventions showed little attempt at between-session interaction, and where this was provided practice exercises were available in learner booklets and so completed individually rather than using ‘interaction’ with others [28]. The inclusion of between session interaction with the facilitator, and more especially with a group network, not only assists through the duration of the intervention but may also provide longer-term benefits through improved connections even after the formal end of an intervention. This was also evidenced through deduction that including a learning mechanism for improved friendship or community connection brings positive intervention outcomes, and in existing reports encouraging intervention innovations to support social connections, stimulate action in communities, and inspire people to take care of their own connections [50]. Neither friendship nor community connections were included as a learning mechanism in any ineffective study. Ehlers [27] found increased social support was directly related to decreased loneliness consistent with the finding that ineffective interventions included neither friendship nor community connection as a learning mechanism. This was reiterated by Kotwal [36] who suggested social experience and flexibility could improve intervention success. This research could be further enhanced by exploring the possibility of an additional effect through incorporation of shared interest groups, which may be more acceptable and impactful to social groups and community connections alone.

Like community connection, behavioural change techniques such as CBT also proved to be an effective learning mechanism. One study sought to expand understanding of the role of psychological interventions in tackling loneliness by comparing internet-based CBT with IPT [34]. While CBT proved effective in reducing loneliness, IPT, which addressed interpersonal psychological processes not directly covered by CBT, was not so, suggesting importance in behavioural as well as psychological process change. The authors however also recognised the lack of prior models and studies, alongside limited prior testing of internet based IPT, may have impacted the validity of IPT’s conceptualisation. Given the proven benefit of CBT, in this and other studies included in this review, more research into related psychological interventions and behavioural change techniques would be beneficial.

### Strengths and limitations

This review provides a detailed analysis of individual intervention characteristics and their effectiveness in the reduction of loneliness for the general population. A narrative synthesis was selected over a meta-analysis given the study aims and high levels of heterogeneity. A strength of this review is that it reports on effective, unclear, and ineffective interventions for loneliness limiting the potential for publication bias. Additionally, a thorough quality assessment was conducted for each of the included studies. As with any review, improvements could have been made to the sensitivity of the search strategy. Including additional terms for ‘intervention’ may have revealed further studies, however we considered this to be at the expense of precision. Furthermore, this limitation was mitigated through the additional screening of citations, abstracts, and reviews. It is also possible that the exclusion of specific population studies in the screening process may have limited the study conclusions. For example, the exclusion of migrant populations as a specific population may have reduced the number of studies included considering minority ethnic groups. Additionally, the exclusion of people with specific health conditions may limit applicability to the most unwell in society. It was however concluded that the nature of this review required the exclusion of specific populations with higher propensity for confounding in loneliness, and that these could be areas for future research. Finally, we note that additional findings may have been lost from the review by the exclusion of non-English language papers.

## Conclusion

This review considered interventions for loneliness with a wide variety of different populations and characteristics. Interventions were predominantly targeted at women of higher education levels, contrary to evidence on the prevalence of loneliness. Thus, further research is warranted considering interventions for male recipients and populations with lower educational levels. Group sessions appeared preferred, however the importance of a person-tailored approach to delivery was also recognised by several included studies. This review also revealed the importance of interaction, particularly through active participation and group or facilitator contact, both during and between sessions. Finally of note, this review found value in considering the intervention period, in particular sustained contact following the conclusion of the intervention to maintain effectiveness. It suggests there is not a ‘quick fix’ to loneliness, but that learnt practices and behaviour should be built into one’s lifestyle to achieve longevity of reduction in loneliness. This was consistent with the observation that aiming to improve friendship or community connection was associated with positive intervention outcomes.

### Electronic supplementary material

Below is the link to the electronic supplementary material.

Supplementary Material 1

## Data Availability

All included studies containing data have been cited in the manuscript. All data generated or analysed during this study are included in this published article, its supplementary information files, or are available from the corresponding author on reasonable request.

## List of abbreviations

CASP: Critical Appraisal Skills Programme
CBT: Cognitive Behavioural Therapy
IPT: Interpersonal Psychotherapy
JBI: Joanna Briggs Institute
RCT: Randomised Controlled Trial

## References

[1] Peplau LA. Loneliness: a sourcebook of current theory, research, and therapy. Volume 36. John Wiley & Sons Inc; 1982.

[2] Leigh-Hunt N (2017). An overview of systematic reviews on the public health consequences of social isolation and loneliness. Public Health.

[3] Holt-Lunstad J, Smith TB, Layton JB (2010). Social relationships and mortality risk: a meta-analytic review. PLoS Med.

[4] Owens J, Sirois F (2019). Review of the impact of loneliness and social isolation on health and well-being and whether people who experience loneliness/social isolation have higher use of public services.

[5] Fulton L, Jupp B. *Investing to tackle loneliness: a discussion paper*. 2015, Social Finance: London. p. 1–36.

[6] McDaid D, Park A, Fernandez J. *Reconnections Evaluation Interim Report* 2016, Personal Social Services Research Unit, London School of Economics and Political Science London. p. 1–36.

[7] Matthews T et al. *Lonely young adults in modern Britain: findings from an epidemiological cohort study*. Psychol Med, 2018: p. 1–10. 10.1017/S0033291718000788 PMC6076992 29684289

[8] Morrish N, Medina-Lara A. Does unemployment lead to greater levels of loneliness? A systematic review. Social Science & Medicine; 2021. p. 114339. 10.1016/j.socscimed.2021.114339 PMC8505794 34455335

[9] Morrish N, Mujica-Mota R, Medina-Lara A (2022). Understanding the effect of loneliness on unemployment: propensity score matching. BMC Public Health.

[10] Michaelson J, Jeffrey K, Abdallah S. *The cost of loneliness to UK employers*. 2017, New Economics Foundation. p. 1–56.

[11] Qualter P (2022). Tackling loneliness evidence review: main report.

[12] Eccles AM, Qualter P (2021). Review: alleviating loneliness in young people – a meta-analysis of interventions. Child Adolesc Mental Health.

[13] Christiansen J et al. *Systematic review and meta-analysis of interventions to reduce loneliness*. PROSPERO, 2020(CRD42020175954).

[14] Bessaha ML (2020). A systematic review of loneliness interventions among non-elderly adults. Clin Soc Work J.

[15] Fakoya OA, McCorry NK, Donnelly M (2020). Loneliness and social isolation interventions for older adults: a scoping review of reviews. BMC Public Health.

[16] Masi CM (2011). A meta-analysis of interventions to reduce loneliness. Personality and Social Psychology Review: An Official Journal of the Society for Personality and Social Psychology Inc.

[17] Moher D (2009). Preferred reporting items for systematic reviews and meta-analyses: the PRISMA statement. PLoS Med.

[18] Wright-St Clair VA (2017). Integrative review of older adult loneliness and social isolation in Aotearoa/New Zealand. Australas J Ageing.

[19] Moola S, Aromataris MZE (2020). Chap. 7: *systematic reviews of etiology and risk* in. JBI Manual for evidence synthesis.

[20] Programme CAS. *CASP Randomised Controlled Trial Checklist [online]*. 2022 [cited 07/07/2022; Available from: https://casp-uk.net/images/checklist/documents/CASP-Randomised-Controlled-Trial-Checklist/CASP-RCT-Checklist-PDF-Fillable-Form.pdf

[21] Popay J et al. *Guidance on the conduct of narrative synthesis in systematic Reviews. A Product from the ESRC Methods Programme. Version 1*. 2006.

[22] Bouwman TE (2017). Does stimulating various coping strategies alleviate loneliness? Results from an online friendship enrichment program. J Soc Pers Relat.

[23] Caputi M, Cugnata F, Brombin C (2021). Theory of mind and loneliness: effects of a conversation-based training at school. Int J Psychol.

[24] Cohen-Mansfield J (2018). Efficacy of the I-SOCIAL intervention for loneliness in old age: lessons from a randomized controlled trial. J Psychiatr Res.

[25] Collins CC, Benedict J (2006). Evaluation of a community-based health promotion program for the elderly: lessons from seniors CAN. Am J Health Promot.

[26] Creswell JD (2012). Mindfulness-based stress reduction training reduces loneliness and pro-inflammatory gene expression in older adults: a small randomized controlled trial. Brain Behav Immun.

[27] Ehlers DK (2017). Regional Brain volumes moderate, but do not mediate, the effects of Group-based Exercise training on reductions in loneliness in older adults. Front Aging Neurosci.

[28] Fields J (2021). In-Home Technology Training among socially isolated older adults: findings from the Tech allies Program. J Appl Gerontol.

[29] Fong P (2021). Evidence that loneliness can be reduced by a whole-of-community intervention to increase neighbourhood identification. Soc Sci Med.

[30] Gaggioli A (2014). Intergenerational Group reminiscence: a potentially effective intervention to Enhance Elderly Psychosocial Wellbeing and to Improve Children’s perception of aging. Educ Gerontol.

[31] Ghanbari N (2021). The effect of coping cat program (CCP) on the loneliness of 8–12 years old children in a primary school of Aliabad Katoul in 2019. Pakistan J Med Health Sci.

[32] Hwang J (2019). Loneliness and social isolation among older adults in a community exercise program: a qualitative study. Aging Ment Health.

[33] Käll A (2020). Lonesome no more? A two-year follow-up of internet-administered cognitive behavioral therapy for loneliness. Internet Interv.

[34] Käll A (2021). Therapist-guided internet-based treatments for loneliness: a Randomized Controlled three-arm trial comparing cognitive behavioral therapy and interpersonal psychotherapy. Psychother Psychosom.

[35] Kanter JW (2021). A brief, Mobile intervention to decrease depression and loneliness and improve relationship quality during the Covid-19 pandemic. J Soc Clin Psychol.

[36] Kotwal AA (2021). A peer intervention reduces loneliness and improves social well-being in low-income older adults: a mixed-methods study. J Am Geriatr Soc.

[37] Kramer LL (2021). Use and Effect of web-based embodied conversational agents for improving eating behavior and decreasing loneliness among Community-Dwelling older adults: protocol for a Randomized Controlled Trial. JMIR Res Protoc.

[38] Larsson E (2016). Effects of a social internet-based intervention programme for older adults: an explorative randomised crossover study. Br J Occup Therapy.

[39] Nazari M (2021). The Effectiveness of Social Participation Educational Program on the feeling of loneliness of Elderly people in Rural areas of Baiza City (South of Fars Province). J Health Sci Surveillance Syst.

[40] Rolandi E et al. *Loneliness and Social Engagement in older adults based in Lombardy during the COVID-19 lockdown: the Long-Term effects of a course on Social networking sites Use*. Int J Environ Res Public Health, 2020. 17(21). 10.3390/ijerph17217912 PMC7662584 33126634

[41] Sandu S (2021). 21st Century Good Neighbor Program: an easily generalizable program to reduce social isolation in older adults. Front Public Health.

[42] Shapira S et al. *Teaching and practicing cognitive-behavioral and mindfulness skills in a web-based platform among older adults through the COVID-19 pandemic: a pilot randomized controlled trial*. Int J Environ Res Public Health, 2021. 18(20). 10.3390/ijerph182010563 PMC8536173 34682309

[43] Steven N, Van Tilburg T (2000). STIMULATING FRIENDSHIP IN LATER LIFE: A STRATEGY FOR REDUCING LONELINESS AMONG OLDER WOMEN. Educ Gerontol.

[44] Russell D, Peplau LA, Ferguson ML (1978). Developing a measure of loneliness. J Pers Assess.

[45] Lubben J (2006). Performance of an abbreviated version of the Lubben Social Network Scale among three European community-dwelling older adult populations. Gerontologist.

[46] de Jong-Gierveld J, Kamphuls F (1985). The development of a rasch-type loneliness scale. Appl Psychol Meas.

[47] Salway S et al. *Reducing loneliness among migrant and ethnic minority people: a participatory evidence synthesis* 2020. 8: p. 10. 32780579

[48] Equality EIfG. *Gender Equality Index 2021: health*. 2021, European Institute for Gender Equality: Luxembourg: Publications Office of the European Union.

[49] Department for Digital C (2020). Media and Sport. Wellbeing and loneliness - community life Survey 2019/20.

[50] Beach B. *Social Connections and Loneliness: Health and Wellbeing Innovation Commission Inquiry*. 2018, International Longevity Centre UK: UK. p. 1–34.

