# Supplementary material for: What works in interventions targeting loneliness: a systematic review of intervention characteristics
Source: BMC Public Health. 2023 Nov 9;23:2214. doi: 10.1186/s12889-023-17097-2 (PMC10636966; doi:10.1186/s12889-023-17097-2)
Supplement: Supplementary file 1 — Supplementary Material 1 [file 12889_2023_17097_MOESM1_ESM.docx]

# Appendix 1 – Critical Appraisal Checklists

## Table A1.1: JBI Checklist for Cohort Studies

| **Author year [reference]** | **QUESTIONS*** | | | | | | | | | | | **Overall Appraisal** |
| --- | --- | --- | --- | --- | --- | --- | --- | --- | --- | --- | --- | --- |
|  | **1** | **2** | **3** | **4** | **5** | **6** | **7** | **8** | **9** | **10** | **11** |  |
| Steven 2000 [42] | CT | Y | Y | CT | N | N | Y | Y | Y | N | Y | Include |
| Collins 2006 [24] | n/a | n/a | Y | CT | N | N | Y | N | Y | N | Y | Include |
| Gaggioli 2014 [29] | n/a | n/a | Y | N | N | N | Y | N | CT | N | Y | Include |
| Bouwman 2017 [21] | Y | Y | Y | CT | N | N | Y | Y | N | N | Y | Include |
| Hwang 2019 [31] | n/a | n/a | CT | N | N | N | Y | CT | CT | N | n/a | Include |
| Rolandi 2020 [39] | Y | Y | Y | CT | N | N | Y | Y | CT | N | Y | Include |
| Caputi 2021 [22] | Y | Y | Y | N | N | N | Y | Y | CT | N | Y | Include |
| Fong 2021 [28] | n/a | n/a | CT | CT | Y | N | CT | Y | N | Y | Y | Include |
| Nazari 2021 [38] | Y | Y | Y | CT | N | N | Y | Y | CT | N | Y | Include |
| Kotwal 2021[ 35] | n/a | n/a | Y | CT | CT | N | Y | Y | N | Y | Y | Include |
| Sandu 2021 [40] | n/a | n/a | Y | CT | N | N | Y | Y | N | N | Y | Include |

Y=Yes; CT=Can’t tell; N=No; n/a=not applicable

*1. Two groups similar and recruited from same population?; 2. Exposures measured similarly to assign to both exposed and unexposed groups?; 3. Exposure measured in valid, reliable way?; 4. Confounding factors identified?; 5. Stated strategies to deal with confounding?; 6. Groups/ participants free of outcome at start of study/moment of exposure?; 7. Outcomes measured in valid, reliable way?; 8. Follow up time reported and sufficient?; 9. Follow up complete? If not were reasons for loss to follow up described and explored?; 10. Utilised strategies to address incomplete follow up?; 11. Appropriate statistical analysis used?

## Table A1.2: CASP Checklist for RCTs

| **Author year [reference]** | **QUESTIONS*** | | | | | | | | | | | | | **Overall Appraisal** |
| --- | --- | --- | --- | --- | --- | --- | --- | --- | --- | --- | --- | --- | --- | --- |
|  | **Section A: Is the basic study design valid for a RCT?** | | | **Section B: Was the study methodologically sound?** | | | | | **Section C: What are the results?** | | | **Section D: Will the results help locally?** | |  |
|  | **1** | **2** | **3** | **4a** | **4b** | **4c** | **5** | **6** | **7** | **8** | **9** | **10** | **11** |  |
| Creswell 2012 [25] | Y | Y | Y | N | CT | Y | Y | Y | Y | SD, SE, p-value | CT | Y | CT | Include |
| Larsson 2016 [37] | Y | Y | Y | N | N | Y | Y | Y | Y | SD, p-value | CT | Y | CT | Include |
| Ehlers 2017 [26] | Y | Y | Y | N | N | N | Y | Y | CT | SD, 95%CI | CT | Y | CT | Include |
| Cohen-Mansfield 2018 [23] | Y | Y | Y | N | N | N | Y | Y | Y | SD, SE | CT | CT | CT | Include |
| Kall 2020 [32] | Y | Y | Y | N | N | N | Y | Y | Y | SD, SE, p-value, 95%CI | CT | Y | CT | Include |
| Ghanbari 2021 [30] | Y | Y | CT | N | N | N | Y | Y | Y | SD | CT | CT | CT | Include |
| Fields 2021 [27] | Y | Y | Y | N | N | N | Y | Y | Y | 95%CI | CT | Y | CT | Include |
| Kall 2021 [33] | Y | Y | Y | N | N | N | Y | Y | Y | SD, SE, p-value, 95%CI | CT | Y | CT | Include |
| Kanter 2021 [34] | Y | Y | Y | N | N | N | Y | Y | Y | SD, SE, p-value, 95%CI | CT | Y | CT | Include |
| Shapira 2021 [41] | Y | Y | CT | N | CT | N | Y | Y | Y | SD, SE, 95% CI | CT | Y | CT | Include |
| Kramer 2022 [36] | Y | Y | Y | N | N | N | Y | Y | Y | SD, p-value | CT | Y | CT | Include |

Y=Yes; CT=Can’t tell; N=No

*1. Study addresses a clearly focussed issue?; 2. Intervention assignment randomised?; 3. Were all participants who entered the study accounted for at its conclusion?; 4a. Participants blinded?; 4b. Investigators blinded?; 4c. People assessing/ analysing outcome blinded?; 5. Study groups similar at start of RCT?; 6. Apart from experimental intervention did study groups receive same level of care?; 7. Were the effects of intervention reported comprehensively?; 8. How precise are the results? (e.g. range/CI); 9. Do intervention benefits outweigh harms and costs?; 10. Can results be applied to the local population?; 11. Would the intervention provide greater value to people in your care than the existing interventions?

# Appendix 2 – Recruitment and data collection

## Table A2: Data Collection

| **Author year [reference]** | **Recruitment method** | **Study type** | **Sample size** | **Time of loneliness assessments** | **Loneliness Measure** | **Comparator** | **Loss to follow up**  **Reasons (n=)** |
| --- | --- | --- | --- | --- | --- | --- | --- |
| **Effective** | | | | | | | |
| Collins 2006  [24] | Newsletters; promotional flyers | Pretest-posttest | T0/T0=339 | T0=baseline;  T1=4-months (last class) | Revised-UCLA (4-item) | NR | Attrition rate <5%. Most common reasons being illness of participant or family member |
| Creswell 2012  [25] | Newspaper advertisements | RCT | T0=40  (I=20, WL=20) | T0=baseline;  T1=8-weeks (end of intervention) | Revised-UCLA (20-item) | Waitlist with no participation in new behavioural health programs during the waiting-period | 15% dropout. I: attended <3 classes (n=4), incomplete post-test assessment (n=1). WL: incomplete post-test assessment (n=1) |
| Gaggioli 2014  [29] | From social senior centres | One-group repeated measures | T0=32 older adults | T0=pre-test;  T1=post-test | Italian loneliness scale (18-item) on 4-point Likert-type scale (1= never, 4=always) grouped into 3 subscales (social, emotional, general) | NR | NR |
| Larsson 2016  [37] | Bulletin boards; newspapers | Randomised crossover | T0=30;  T1/T2=28 | T0=initial pre-test;  T1=3-months in  T2=following final intervention period | UCLA (Swedish version) | Participants split into two groups: group 1 = intervention period/control period, group 2 = control period/ intervention period | Discontinued intervention (n=2) |
| Ehlers 2017  [26] | NR | RCT | T0=247;  T1=169 | T0=baseline;  T1=post-intervention | UCLA (20-item) | Comparison made across the four exercise interventions | Did not receive allocated intervention (n=1), lost to follow up (n=38), MRI data quality/abnormality (n=39) |
| Bouwman 2017  [21] | Online community website advert; regional newspapers | Intervention | T0=239;  T1=120;  T2=80;  T3=67 | T0=baseline;  T1=after 5 lessons; T2=end of program;  T3=after 1 year | De Jong Gierveld (11-item); direct question 'How did you feel today?' on 7-point scale from 'lonely' to 'not lonely' | Light alternative with limited information and reflection, and no assignments | Participants lost to follow up did not differ in social or emotional loneliness, gender, self-rated health, having a partner, having children. Participants who dropped out were slightly younger (mean age 60.7) than those remaining |
| Cohen-Mansfield 2018  [23] | Health Maintenance Organization; phone calls with local older people on a commercial vendor list; university lectures; local senior centres; posters; local older person residential buildings; referral from other studies, other participants, or social services | RCT | T0=89 (I=45, C=44);  T1=74 (I=39, C=35);  T2=63 (I=35, C=28) | T0=baseline;  T1=post-intervention;  T2=3-month follow-up | UCLA (8-item); direct question ‘how often would you say you feel lonely?’ on 6-point scale from ’never’ to ’several times an hour’; direct question ‘to what extent do you feel lonely?’ on 5-point scale from ‘not at all’ to ’to a very large extent’ | Control group received no social engagement nor help in promoting engagement from research staff. | I: health (n=2), not wish to continue (n=4), cognitive impairment (n=1), could not continue (n=1). C: could not be reached (n=1), could not continue (n=3), health (n=3), not wish to continue (n=8), other (n=1) |
| Hwang 2019  [31] | Random selection from database of ‘Walk n Talk’ program participants | Qualitative interviews | T0=16 | T0=initial;  T1=final | Lubben Social Network Scale (6-item); UCLA (20-item); de Jong Gierveld (11-item) | None | n/a |
| Kall 2020  [32] | Newspaper advert; social media; posters | Pre-treatment post-treatment randomised trial | T0=73 (I=36, C=37);  T1=61 (I=27, C=34);  T2=44 (I=21, C=23) | T0=pre-treatment;  T1=post-treatment;  T2=2-year follow-up | UCLA version 3 (20-item) | Waitlist group received access 2-weeks after the initial treatment period. Contacted therapists themselves if need arose (guidance-on-demand) | Declined participation during treatment period (n=7), lost to follow up (n=22). 41% missing data at 2-year follow-up for UCLA-LS-3. No significant difference between completers or non-completers |
| Ghanbari 2021  [30] | Convenience sampling from participating primary school | Randomised interventional | T0=60 (I=30, C=30) | T0=before intervention;  T1=after intervention | Usher’s loneliness questionnaire (24-item) on 5-point Likert scale (1=not true at all, 5=absolutely true) with higher score indicating greater loneliness | No action taken for the control group. Educational materials were provided after the study. | NR |
| Fong 2021  [28] | Newspapers; community space notices; national and social media; ‘Relationships Australia’s’ website | Prospective three-point longitudinal survey | T0=437;  T1=207;  T2=196 (6-months after T2) | T0=month before neighbour day;  T1=month following neighbour day | Single item question  Rate extent agree with the statement ‘I often feel very lonely’ (scale 1-7) | NR | Participants who felt more lonely at T0 were significantly more likely to complete the survey at T1. Participants completing T2 surveys did not differ significantly on T0 loneliness score |
| Kall 2021*  [33] | Social media; posters; primary care and student health centre contacts; newspaper articles | RCT | T0=170 (IPT=68, WL=34, CBT=68);  T1=130 (IPT=57, WL=27, CBT=46);  T1 intention-to-treat=170 (IPT=68, WL=34, CBT=68);  T2 IPT=42, CBT=40 | T0=before treatment; T1=post-treatment; T2=4-months post-intervention.  Also, every 2-weeks during intervention | UCLA version 3 (20-item) | Waitlist received treatment after 11 weeks and could contact the research group via email if needed. Control group interaction was infrequent and handled by a different person to the treatment group contact | To T1: discontinued intervention (n=14); lost to follow-up (n=40). To T2: lost to follow-up (n=54) |
| Nazari 2021  [38] | Rural health centres | Quasi-experimental interventional | T1=239  (I=119, C=120) | T0=pre-intervention;  T1=1-month post-intervention | UCLA (20-item) | No educational intervention provided to control group and no information transferred from intervention to control group | NR |
| Kotwal 2021  [35] | Via an urban senior centre. Staff presented to partners at places seniors gather | Mixed methods - longitudinal study and interviews | Quantitative n=74; qualitative interviews n=15 | T0=baseline;  T1=6-months;  T2=12-months;  T3=18-months;  T4=24-months | UCLA (3-item) | None | Loss to follow-up (n=22), COVID-19 shelter-in-place orders (n=19), other (n=13) |
| **Can’t tell if effective** | | | | | | | |
| Steven 2000  [42] | Local newspapers, folders distributed in older person neighbourhood centres | Follow-up study | T1/T2=32 | T1=immediately following course;  T2=1 year later | de Jong Gierveld (11-item) | Control group drawn from a national longitudinal survey employing a representative sample of women who had not participated in the course | None |
| Rolandi 2020  [39] | NR | Pre-post intervention | T1=130  (trained=60, untrained=70) | T0=pre-intervention; T1=post-intervention; T2=1-year after post-intervention evaluation | UCLA (3-item) | Waiting list (inactive control group) or a lifestyle education course (active control group) | Death (n=1), refusal (n=4), unreachable (n=9) |
| Caputi 2021  [22] | Local primary school enrolment and parental consent | Pre-post intervention | T0=210  (I=105, C=105) | T0=start of school year;  T1=1-week post-intervention;  T2=2-months post-intervention | Loneliness and Social Dissatisfaction Questionnaire (24-item) | No-Theory of Mind group presented with 10 physical stories with trainer feedback to explain reasons their answers were right or wrong | NR |
| Kanter 2021  [34] | News media; social media adverts; smartphone research platform | RCT | T0=1420  (I=719, C=701); enough data for loneliness outcome analysis = 1242 | T0=baseline;  T1=day 22 (intervention);  T2=day 28 (post-intervention) | Two items adapted from UCLA Loneliness Scale “I felt lonely today” and “I felt left out today” | NR | No responses after T0 (n=81), no response after intervention began on day 6 (n=96) |
| Shapira 2021  [41] | Online invitation circulated in WhatsApp groups | RCT | T0=82  (I=64; C=18) | T0=baseline; T1=immediately post-intervention;  T2=1-month follow-up | UCLA (3-item) | Waitlist control receiving access to the intervention within 4 weeks | I: health reasons (n=5), lack of interest (n=5), technical difficulties (n=3)  C: lack of interest (n=6), non-response (n=3)^Ϯ^ |
| **Not effective** | | | | | | | |
| Fields 2021  [27] | By phone from the network of a volunteer-based organisation | Randomised pragmatic pilot trial | T0=70  (I=38, WL=32);  T1=57  (I=27, WL=30) | T0=baseline;  T1=2-months (I=post-training survey, C=2-month survey) | UCLA (3-item) dichotomised to lonely when reporting ‘often’ or ‘some of the time’ to any of the three scale items | 2-month waitlist group | I: lost interest (n=2), unable to contact (n=3), internet cost (n=3), dropped out (n=1), not a good time (n=1), living conditions unsuitable for volunteers (n=1), health (n=2), relocation (n=1), declined post-training survey (n=1), other (n=2). WL: dropped out (n=2), health (n=6), unable to contact (n=1), not a good time (n=2), lost interest (n=3), language barrier (n=1), death (n=1), refused home internet (n=1), volunteer rematch delays (n=1), dissatisfaction with volunteer (n=1) |
| Sandu 2021  [40] | Mailed postcard information to older adults served by a community service agency | Observational cohort study | T0=1361;  intervention engagement=261; baseline UCLA=141; at least 1 UCLA follow-up=84 | T0=baseline;  T1=first year of follow up | UCLA (3-item) | NR | NR |
| Kramer 2022  [36] | Research panels; flyers; newspapers; social media | RCT | 51 met inclusion criteria, 32 used in analysis | Tw=joining waitlist (4-weeks before T1);  T0=create account;  T1=4-weeks;  T2=8-weeks | De Jong Gierveld (6-item) | Control received intervention access after a 4-week waiting list. | No response (n=7), illness (n=3), lack of time (n=3), lack of motivation (n=3), difficulties with the service (n=2), internet issues (n=1) |

I=intervention; C=control; WL=waitlist; NR =not reported.

CBT=cognitive behavioural therapy; IPT=interpersonal psychotherapy.

Ϯ Kall 2020b ‘Internet-based…’

* Kall 2021 included an effective CBT intervention, though also included an ineffective IPT intervention.

# Appendix 3 – Intervention Effectiveness

## Table A3: Intervention statistical results and key conclusions

| **Author year [reference]** | **Mean (SD)** | | | **Additional analysis and models** | **Key conclusions** |
| --- | --- | --- | --- | --- | --- |
|  | **T0** | **T1** | **T2** |  |  |
| **Effective** | | | | | |
| Collins 2006  [24] | 8.64 (0.10) | 7.86 (0.09) | - | t=-9.20, p<0.001 | The community based educational program was found to reduce loneliness. Minority participants with low incomes or with higher education levels showed greatest reduction in loneliness suggesting the program may have greatest impact on individuals with higher risk of health problems |
| Creswell 2012  [25] | I: 42.35 (2.23)  WL: 38.40 (2.33) | I: 37.40 (2.51)  WL: 40.75 (2.30) | - | Mixed effect linear model= significant treatment condition x time interaction (F(1,35)=7.86, p=0.008). Follow-up ANCOVA with complete pre-post data (N=33): Intervention (M=35.65, SE=2.02); Waitlist (M=42.33, SE=1.73), thus intervention had lower post-test loneliness levels after controlling for baseline loneliness (F(1,30)=6.27, p=0.02) | The intervention provides promise as a novel approach for reducing perceptions of loneliness in older adults. Future RCT studies should use these initial findings in larger samples with active control groups |
| Gaggioli 2014  [29] | Emotional: 1.76(0.48)  Social: 3.24(0.65)  General:  1.88(0.51) | Emotional:  1.60(0.46)  Social:  3.25(0.47)  General:  1.68(0.51) | - | t-student for pre-post intervention comparisons: emotional loneliness t=2.074*; social loneliness t=-0.55; general loneliness t=2.195* | The intervention contributed to a significant decrease in participants' feelings of general and emotional loneliness |
| Larsson 2016  [37] | G1:45.53 (7.41)  G2: 49.93 (8.61) | G1: 42.43 (7.44)  G2: 41.93 (8.82) | G1: 42.00 (7.34)  G2: 39.50 (10.42) | %change Group 1 [Group 2]: T2-T1=-0.07%(0.07) [-0.05%(0.09)]; T3-T1=-0.08%(0.08) [-0.09%(0.13)]. Interaction between intervention and sequence (I/C vs C/I) F=0.755, p=0.4765. Time effect among T1, T2, T3 F=13.156, p<0.001. Wilcoxon’s signed-rank test revealed significant pre- post-intervention reduction in loneliness G1 p=0.003, G2 p=0.049. Intervention effects sustained 3-months post intervention for G1 with no significant changes detected for the already established effects | The programme has potential to reduce experiences of loneliness. This study adds knowledge about client-centred, individually adapted interventions and the need for such programmes in a more technology-based society |
| Ehlers 2017  [26] | 37.12 (9.77) | 35.31 (8.91) | - | Change p<0.01. Latent construct variance at baseline=0.63*. Mean latent change score=-0.15*, bias corrected 95%CI[-0.23,-0.08], variance=0.44*, standardised effect size d=-0.19 | Exercise programs may be effective in reducing loneliness in older adults, regardless of exercise mode. Direct effects can arise through increases in social support and may also be indirect through decreases in perceived stress |
| Bouwman 2017  [21] | Full [light]  Social: 3.67[3.71]  Emotional:  4.45[4.26] | Full [light]  Social: 3.39[3.85]  Emotional:  4.03[4.48] | Full [light]  Social:  3.10[3.17]  Emotional:  3.82[4.03] | T4 mean loneliness full [light] social=2.83[3.16], emotional=3.33[3.06]. Full group [light alternative] baseline, week 5, week 10, 1 year, respectively B(SE): linear regress social loneliness on time in intervention 3.67(0.15***), -0.34(0.15*), -0.53(0.17**), -0.70(0.20***) [3.71(0.15***), -0.11(0.18), -0.76(0.21**), -0.50(0.21*); linear regress emotional loneliness on time in intervention 5.55(0.16***), -0.40(0.17*), -0.49(0.19*), -0.86(0.22***) [4.26(0.18***), -0.02(0.18), -0.36(0.21), -0.99(0.21***)]. Linear regression of baseline loneliness and demographics on loneliness directly after the program B(SE), n=80: social loneliness, range 0-5, constant 1.84(1.88), baseline social loneliness 0.84(0.13***); emotional loneliness, range 0-6, constant 2.44(1.70), baseline emotional loneliness 0.73(0.09***) | Loneliness intensity decreases after baseline for both full and light interventions, however many participants still experience some loneliness after the course |
| Cohen-Mansfield 2018  [23] | I: n=39 3.08(0.73)  n=35 3.05(0.74)  C: n=35  2.94(0.98)  n=28  2.92(1.05) | I: n=39 2.81(0.74)  C: n=35  3.06(0.89) | I: n=35 2.72(0.67)  C: n=28  2.92(0.88) | Significant interaction terms in both ANOVAs comparing intervention and control after the intervention (F_(1,72)_=4.95, p<0.05) and after the follow-up period (F_(1,61)_=4.38, p<0.05) | The intervention significantly decreased loneliness as compared to a no treatment control group, though effect size is modest. Reflect that the intervention period was too short, something voiced by most participants |
| Hwang 2019  [31] | - | - | - | Qualitative interviews revealed the program helped decrease perceived loneliness feelings by making new social connections, generating sense of belonging, improving health and well-being. Lubben measures of social isolation did not change significantly. Significant decrease in loneliness observed on UCLA loneliness score and the de Jong-Gierveld emotional loneliness score | Intervention participants reduced their feelings of loneliness by promoting sense of belonging through socialising and exercise programs |
| Kall 2020 ^Ϯ^  [32] | I: 58.61 (4.15)  C: 59.62 (7.47) | I: 50.52 (6.95)  C: 56.24 (9.41) | I: 47.57 (8.48)  C: 48.23 (12.99) | Cohen's d observed mean effect sizes: intervention pre-treatment to follow-up d=1.65[1.02, 2.29]; control/wait list pre-treatment to follow up d=1.07[0.5, 1.65]; effect size between original treatment group and the waitlist/guidance-on-demand group at follow-up d=0.06[-0.56, 0.68]. Piecewise mixed model indicated significant reduction in loneliness for whole sample during follow-up: b=-7.96 [-11.70, -4.23], SE=1.88, p=0.0001. Result significantly steeper in guidance on demand (control) group during follow-up after the waiting period b=5.77 [0.32, 11.23], SE=2.75, p=0.0382. 59.1% (n=22) of participants completing loneliness at follow-up indicated reliable change from pre-treatment to follow-up, while 1 participant (2.3%) was classified as reliably deteriorated | Significant reduction in loneliness for entire sample, particularly guidance-on-demand (waitlist). Results support the use of CBT-interventions targeting loneliness, and specifically the potential for internet interventions in relieving loneliness. Effects of internet-based CBT can be enduring |
| Ghanbari 2021  [30] | I: 66.66 (5.52)  C: 70.00 (9.82) | I: 62.03 (9.71)  C: 69.71 (12.23) | - | Intervention p-value 0.03; control p-value=0.92. Independent t-test showed no significant difference between intervention and control groups before intervention at T1 (p=0.11), however there was a significant difference after the intervention at T2 (p<0.01). | The intervention had a positive and significant effect on loneliness in children. Loneliness score decreased significantly compared to pre-test score, and to the control group |
| Fong 2021  [28] | - | - | - | Results of linear mixed modelling predicting T2 loneliness, β(SE). Model 1-inc. postcode=3.06***(0.12). Model 2–inc. baseline loneliness and neighbourhood identification=1.37*(0.58); T1 loneliness=0.63***(0.06). Model 3–inc. T2 neighbourhood identification=2.10***(0.64); T1 loneliness=0.62***(0.06). Model 4–inc. covariates age, sex, education, SES =2.82***(0.76); T1 loneliness=0.61***(0.06) | Participation in Neighbour Day increased neighbourhood identification which in turn reduced loneliness and increased social cohesion. Reduced loneliness at T2 then predicted improved wellbeing at T3 |
| Kall 2021^φ^  [33] | CBT: 57.59 (6.80)  IPT: 58.46 (7.67)  WL: 59.18 (8.00) | CBT: 48.37 (9.30)  IPT: 53.53 (7.98)  WL: 57.07 (7.53) | CBT: 50.10 (8.28)  IPT: 52.43 (10.44) | CBT significantly favoured over waitlist during the treatment (b=-5.22, 99%CI[-9.61, -0.83], SE=1.70, p=0.006, d=0.71). IPT did not significantly decrease loneliness relative to waitlist during treatment period (b=-1.36, 99%CI [-4.19, 1.48], SE=1.44, p=1, d=0.18). CBT had significantly larger reduction in loneliness compared to IIPT (b=-3.87, 99%CI[-7.28, -0.45], SE=1.32, p=0.012, d=0.53). Gains maintained at 4-month follow-up, however non-significant increase in loneliness in ICBT group (b=1.66, 95%CI[-0.57, 3.88], SE=1.14, p=0.144) and nonsignificant decrease for IPT group (b=-1.25, 95%CI[-2.97, 0.47], SE=0.88, p=0.155) | ICBT led to significant reduction in loneliness compared to waitlist, while IIPT did not significantly reduce loneliness. The effect was maintained but not improved at 4-months follow-up |
| Nazari 2021  [38] | I: 62.24 (7.53)  C: 49.30 (13.15) | I: 28.86 (6.88)  C: 51.26 (11.82) | - | Paired t-test: intervention=0.001; control=0.07. Independent samples t-test: pre-intervention=0.66, post-intervention=0.001 being a significant difference between intervention and control after the study | Significant relationship between social participation and loneliness. Educational program of social participation can be used to plan interventions for predicting, improving and modifying feelings of loneliness |
| Kotwal 2021  [35] | 3.4 | 2.9 | 2.5 | T3=2.6, T4=2.6 loneliness score. Score on range 0-6: 0=none, 1-2=moderate, 3-6=high. Loneliness scores decreased by 0.8 points on average (p=0.015). Most of the change in loneliness occurred in the first 12 months, and sustained at 24 months | Program associated with sustained, reduced feelings of loneliness. The first to demonstrate a successful peer support program amongst diverse, low-income adults |
| **Can’t tell if effective** | | | | | |
| Steven 2000  [42] | I: 7.2 (2.9)  C: 7.1 (3.1) | I: 4.5 (3.2)  C: 5.5 (3.7) | - | Intervention t=5.43, p=0.000, control t=4.25, 0=0.000. Mean change score for program participants =-2.59(2.89) is significantly greater than for control group =-1.41(1.8) t=-1.96, p=0.054). At statistical significance of at least p<0.05 in intervention group 11/32(34%) reduced loneliness, 2/32(6.2%) increased loneliness; in control group 7/32(21.9%) reduced loneliness, none increased | Significant reduction in loneliness observed in both intervention and control groups, authors suggest could be due to high baseline loneliness or measurement errors. Potential problem of self-selection meaning not possible to conclude whether the friendship program would work for all lonely older women |
| Rolandi 2020  [39] | - | - | - | Non-significant differences were found for the UCLA loneliness scale total score. UCLA total score: cross sectional analysis mean(SD) trained = 4.2(1.5), untrained = 4.6(1.6), p=0.124; longitudinal analysis mean(SE) trained = 0.0(0.2), untrained = 0.3(0.2), GLM p=0.135 | Non-significant differences were found for the UCLA loneliness scale total score. Social network site training showed benefits in specific loneliness feelings (UCLA item ‘feeling left out’) during the COVID-19 lockdown isolation period |
| Caputi 2021  [22] | ToM: 29.12 (10.06)  No-ToM:  29.07 (9.85) | ToM: 27.42 (9.34)  No-ToM:  28.17 (9.01) | ToM: 28.56 (10.13)  No-ToM:  28.66 (9.38) | Linear mixed effects model value(std.error): intercept=3.45***(0.08), vocabulary T1=-0.01*(0.00), ToM T1=0.05(0.05), ToM T2=-0.08**(0.03), ToM T3=-0.05(0.03), no-ToM T2=0.00(0.03), no-ToM T3=-0.00(0.03) | Loneliness score significantly decreased post-test for children attending intervention, compared to control. ToM may have changed group dynamics, thoughts and behaviours producing more prosocial, inclusive, activities. Conversation-based ToM training was beneficial in the short- but not long-term as not significant 2 months post-intervention |
| Kanter 2021  [34] | I: -0.07 (0.11)  C: -0.07 (0.11) | I: -0.15 (0.11)  C: -0.08 (0.11) | I: -0.13 (0.11)  C: -0.13 (0.11) | Treatment group= lower loneliness (intervention effect = 0.07, 95% CI = 0.14 to 0.01, p = 0.031) not significant on the last day of the one-week follow-up period (intervention effect = 0.00, 95% CI = −0.09 to 0.09, p = 0.934) | The mobile based intervention had a significant, but not lasting, effect on loneliness during the COVID 19 pandemic. Intervention effects were strongest on the last day but stopped growing once the intervention ended and were no longer significant after 1-week follow-up |
| Shapira 2021  [41] | I: 5.4 (2.0)  WL: 6.3 (1.9) | I: 4.7 (1.6)  WL: 6.2 (1.7) | I: 5.0 (1.8) | Intervention difference T0 to T1 M(SE)=-0.7(0.28) t(63)=2.15*; difference T1 to T2 M(SE)=0.3(0.26) t(63)=-0.88; difference T2 to T3 M(SE)=-0.4(0.24) t(63)=1.5. I.e., gains from T1 not maintained at T2 (p>0.05). Waitlist control difference T0 to T1 M(SE)=-0.1(0.32) t(17)=0.34. No changes in loneliness found in waitlist control group. ANOVA showed significant main effect of time-by-group interaction (F(1, 78)=5.59, p=0.02, h2=0.07) indicating groups differed in loneliness post-intervention. Main effect of time did not reach statistical significance | Intervention saw decrease in loneliness from T0 to T1 suggesting regular contact was helpful in alleviating at least some loneliness during the pandemic, strengthening the notion that digital group interventions are effective. Social interventions may only be helpful in reducing loneliness when they are sustained over time |
| **Not effective** | | | | | |
| Fields 2021  [27] | - | - | - | A) Quantitative findings. Intervention: T1=92% lonely, T2=88% lonely. Waitlist: T1=67%, T2=66%. Loneliness outcome adjusted for baseline loneliness = adjusted OR=1.37[95%CI: 0.10, 15.62]. Overall no quantitative change in loneliness from survey data. B) Qualitative findings. Loneliness was a central driver of program participation | No quantitative changes in loneliness were observed |
| Sandu 2021  [40] | 4.26 (1.41) | 4.15 (1.41) | - | Did not complete follow up[n=57] mean(SD)=4.16(1.00). No significant change in the UCLA loneliness score in the first year of follow up | The program expanded the social network of participants however no significant change in loneliness score was found |
| Kramer 2022  (36) | 2.47 (1.78) | 2.62 (1.91) | 2.44 (1.92) | Waitlist questionnaire = 2.27(1.71). No decrease in loneliness (χ2=0.02, p=0.99) | No reduction in loneliness. It may be that the expected decrease in feelings of loneliness were counteracted by increasing loneliness in the target group during the Covid-19 pandemic |

*p<0.05, **p<0.01, ***p<0.001

I=intervention; C=control; WL=waitlist

G1=group1; G2=group2

CBT=cognitive behavioural therapy; IPT=interpersonal psychotherapy; ToM=Theory of Mind

^Ϯ^ Kall 2020 included as effective given the intervention (resource access and facilitator contact) had been received by both groups (intervention and waitlist control) by the time of follow-up loneliness measurement

^Φ^ Kall 2021 included an effective CBT intervention, though also included an ineffective IPT intervention

# Appendix 4 – Intervention Characteristics

## Table A4: Detail of intervention characteristics

| **Author year [reference]** | **Number of sessions / intervention duration** | **Session duration** | **Group / individual** | **Online / in person** | **Tasks / structure** | **Between session practice?** | **Other information** |
| --- | --- | --- | --- | --- | --- | --- | --- |
| **Effective** | | | | | | | |
| Collins 2006  [24] | 16-weeks. Classes taught weekly totalling an average of 32 hours instruction per participant | 2 hours | Group | In person | Primarily delivered in senior centres and senior housing developments by paraprofessionals, volunteer peer educators and on-site staff. Curriculum taught in an interactive style that promotes participation | Participants encouraged to integrate one new idea/skill from each lesson | Educational health promotion intervention. Session topics include nutrition and food, personal safety, financial strategies, wellness, and productive aging |
| Creswell 2012  [25] | 8-weeks of weekly sessions with a retreat in week 6 or 7 | 120-min sessions. Day-long retreat | Group sessions directed by trained clinician instructors | In person | Guided mindfulness meditation exercises, mindful yoga and stretching, and group discussions. Day long retreat sought to integrate and elaborate exercises from prior sessions | 30-mins of daily home mindfulness practice 6 days a week | Mindfulness-Based Stress Reduction intervention. Administered over 3 cohorts |
| Gaggioli 2014  [29] | Met once a week for 3-weeks | 2 hours | Elderly participants assigned to small senior-student groups (2 older adults, 6-8 children) facilitated by psychologist | In person | Session 1: briefed on activity goals and program structure, told most interesting reminiscences would go on the project website, elderly encouraged to share life experiences, children to ask questions. Session 2: elderly shared memories while integrating material/documents from around their home, encouraged to focus on reminiscences where children showed most interest, children encouraged to take notes, ask questions, make comments. Session 3: participants collaborated to select most interesting reminiscences and reported their content in written form, selected stories uploaded to project website, debriefed on what liked or disliked | NR | Elderly encouraged to share memories and promote pupil interaction |
| Larsson 2016  [37] | 34-weeks. Individual meetings offered weekly with frequency and type of support (in home/remote) adapted to participants’ needs. Group meetings offered every second week | Max 1.5hrs | Individual and group meetings. Intervention provided by part-time occupational therapists. | Online and in person | Programme includes a description of the time, place and content. Programme content included pre-decided actions for occupational therapists (e.g. establishing collaborative relationships). Participants have pre-decided tasks and individually adapted goal-directed tasks | Individual meetings offered more frequently if needed with additional support available to accomplish programme tasks | Social internet-based intervention combining, including in-home and remote support via internet or telephone. Therapists had experience working with older adults and 2-days specific intervention delivery training. They worked in parallel, responsible for six to eight participants each per intervention period |
| Ehlers 2017  [26] | 24-weeks. 3 exercise sessions per week | 1-hour | Group sessions supervised by trained exercise leaders | In person | Sessions began with brief warmup of walking and stretching and concluded with a set of stretches. Participants randomly assigned to one of four interventions: dance (aerobic + cognitive training), strength/stretching/stability (active, non-aerobic control), walk (aerobic), and walk plus (aerobic). Dance and strength training involved learning new exercises each month with increasing speed and complexity | NR | NR |
| Bouwman 2017  [21] | 6-week course | NR | Individual | Self-guided online program | Lessons involve reading informative text and facilitate reflection through questions and animated videos. Participants were asked to imagine a specific situation and think about how they would react | NR | Covers topics on making and maintaining friendships. Invited to access new lessons weekly by email. Once completed, lessons remain available |
| Cohen-Mansfield 2018  [23] | Up to 10 individual meetings; up to 7 group sessions. Mean 4 [range 0-7] group sessions, 5 [range 1-13] personal meetings attended | NR | Participants chose group, individual or both session types. All formats included an activities counsellor. Groups also include other participants | In person | Individual meetings address personal barriers to social integration, options for social contacts, and techniques and local resources to tackle barriers.  Group sessions provide opportunities to practice skills, discuss barriers and ways to address them | NR | Aimed to identify barriers for the specific person |
| Hwang 2019  [31] | Sessions twice a week for 12-weeks | 2hrs 35mins | Group | In person | 45-min fitness program consisting of weight and strength training designed to reduce falls, 30-min walk group, 60-min interactive health education session (participant chosen topics), and 20-min open socialisation. Education session speakers included community professionals and volunteers | NR | Held in local community centres and seniors' residence buildings.  Participants coached to 'go at their own speed and ability' |
| Kall 2020  [32] | 8 modules delivered over 8 weeks | NR | Individual. Guided through the programme by a therapist (final year clinical psychology student). | Online | Modules contained psychoeducational text and picture elements conceptualised in cognitive-behavioural therapy terms and surrounding the theme of loneliness. Participants received a weekly introductory message from the therapists. Modules unlocked one at a time on a weekly basis regardless of the completion status in the previous module. Once treatment concluded participants had no more contact with their therapist nor access to the modules | Participants given homework assignments to complete during the week. Therapists could be contacted between sessions if need arose | Programme was based on behavioural activation and cognitive restructuring. Therapists provide feedback within 24 hours |
| Ghanbari 2021  [30] | 4 times a week for 4 weeks (16 sessions in total) | 30 mins | NR | In person | Week 1 (sessions 1-3) focussed on shaping direction of treatment, identifying feelings and physical responses to anxiety and loneliness. Week 2 (sessions 5-8) included relaxation training and self-coping strategies/skills. Sessions 4 and 9 involve a meeting with parents to encourage their involvement in the treatment program. Weeks 3 and 4 (sessions 10-16) involved exercises in situations where would have different levels of loneliness and anxiety to practice coping skills | NR | Brief explanation provided prior to start of intervention |
| Fong 2021  [28] | One specific day of the year | 1 day | Individuals and community groups | In person and online | Resources could be found on Relationships Australia's website to help plan and host events. Events included social gatherings, cards in letterboxes, checking in on vulnerable neighbours, community-based games, community meetings, social media | NR | Nationwide campaign 'Neighbour Day' where the public are encouraged to connect with others in their neighbourhood on a specific day of the year |
| Kall 2021*  [33] | 9 modules delivered over 9 weeks | NR | Individual | Online | Each online intervention (IPT and CBT) had 9 modules containing text, pictures, and interactive assignments related to loneliness and how to deal with it. All participants received weekly feedback on their work from their assigned therapist. No limit to the amount of therapist contact | Therapists could be contacted between sessions if need arose | Could contact therapists through the platform's messaging service and receive response within 24hrs on workdays |
| Nazari 2021  [38] | Once a week for 5 weeks (5 sessions total) | 60-80 mins | Group | In person | Techniques included interactive lecture, group discussion, description of personal experiences, short recreational tours. In each session the researcher used verbal encouragement, emotional arousal, emotion-expressing techniques, previous successful experiences, lecture, and question and- answer methods. Shared experiences used to help each other and for self-help groups.  Session 1 aimed to increase interest and self-efficacy about definition, causes and clinical signs. The session started with participant introductions and ended with a summary and participants asked to contemplate the question “which tasks reduce their loneliness?” before the next session. In session 2 sought to empower people using emotion-expressing techniques and develop optimism and positive thinking. Session concluded with participants sharing their experiences to reduce feelings of loneliness. Session 3 devoted to avoiding disturbing thoughts, prioritising social participation, increasing participation in religious rituals. After session participants were encouraged to perform religious ceremonies. Session 4 aimed to increase social intimacy with participants taken to recreational public places to improve social interactions alongside by a walking group and sharing breakfast together. Session 5 was held outdoors and participants shared their experiences associated to goals, achievements, and barriers of the program | Any action to form group meetings was encouraged, as was verbal social participation and through gifts | Sessions aimed for participants to reach a common understanding of loneliness with a common goal of confronting it. Designed to enhance learning about causes and consequences of loneliness with social participation as a suitable solution. Aimed to introduce social activity on a gradual basis and share how obstacles were overcome |
| Kotwal 2021  [35] | Between 6- and 24-months | NR | Individual and/or group | In person | Peers began with a “soft approach” of home visits, providing companionship for simple errands, or connecting individuals to city services. Additional subsequent social activities might include shared meals, group activities, art programs, or walks around the city. Larger monthly cultural events held in community venues with multiple participants were occasionally organised by peers | Social activities as rapport grew | NR |
| **Can’t tell if effective** | | | | | | | |
| Steven 2000  [42] | 12 lessons | NR | Group | In person | Lessons focused on topics related to friendship, such as: expectations, early experiences, self-evaluation as a friend, making new friends, improving existing friendships, setting friendship goals. Each lesson included: theory; practicing skills important in friendship, e.g. listening, self-disclosure, empathy, assertiveness; role play of difficult social situations; homework assignment. Participants can also discuss personal experiences related to the topic | Participants completed a homework assignment | Control group matched on baseline loneliness and background variables |
| Rolandi 2020  [39] | Five sessions held twice a week, followed by face-to-face tutoring available twice a week for 3-weeks | 2 hours^φ^ | Group | In person | Interactive groups sessions. Themes included: smartphone use, Facebook and WhatsApp use, privacy rules, fraud risk prevention using Facebook. Participants provided with user-friendly smartphones designed for older adults | Messages and media regularly sent in a dedicated WhatsApp group and Facebook pages | NR |
| Caputi 2021  [22] | Five, weekly, sessions during school-time | 50-mins | Group session delivered by 4 specifically trained psychology interns | In person | All children presented with a written story and asked to individually answer the story questions on paper. Sessions included group discussion about two stories each with a language exercise. Experimenter highlights the core message for each story and children are asked to recall an episode similar to the one in the story. ToM presented with 10 mentalistic stories with trainer feedback to expand children's comments and facilitate understanding of different perspectives | NR | NR |
| Kanter 2021  [34] | 16 days (days 7-22) | Shortest activity took approx. 5 mins, longest approx. 10–12 minutes | Individual | Online. Mobile based | Introductory message on day 7. At 8:00am (days 8-22) participants received one of 14 suggestions. All suggestions encouraged participants to engage in specific practices, of differing lengths, during the day. The simplest ones (e.g., Gratitude and Loving Kindness Meditation) only included text messages and a brief audio file delivered via text; the more complex included text messages and a web page link which included text or embedded audio files describing why a suggestion was being made, how to engage in the practice, and audiotaped exchanges between team members describing what it was like to try the practices themselves. | NR | Day 7 message alerted participants to expect their first suggestion via text message the next morning.  Some suggestions included additional text reminders at 12pm and 4pm. |
| Shapira 2021  [41] | 7 sessions over 3.5 weeks | 1-1.5 hours | Group sessions delivered by 4 moderators (trained clinical social workers) | Online | Online Zoom videoconferencing sessions delivered to groups of 5-7 people. Two main components of the sessions were a) guided group discussions, b) learning and practicing cognitive-behavioural techniques and skills (e.g., breathing, guided imagery of a ‘safe place’, constructing positive self-talk, mindfulness meditations) aimed to identify non-adaptive cognitive schemas and promote better coping | WhatsApp group (including moderator) to facilitate between session discussion and practice | WhatsApp was also used as a platform for learning by sending supplementary materials and practicing techniques |
| **Not effective** | | | | | | | |
| Fields 2021  [27] | Eight weekly sessions | NR | Individual 1:1 digital training sessions | Online | Sessions covered: getting to know the iPad, using the iPad, online safety, email, email safety, communicating via apps and FaceTime, online communities, having fun and wrap-up. Participants had learner booklets outlining curriculum topics and including step-by-step visual guides and practice exercises. Participants each received a tablet, tablet case, stylus, broadband access, and certificate of completion | Practice exercises provided in the learner booklets | Incorporated into an existing volunteer based friendly visitor program. Internet connection paid for during study after which participants could continue the service for $10 per month. Could keep tablet and accessories at no cost |
| Sandu 2021  [40] | 1 year | 11 min median phone call length | Individual | Over the phone | The first call obtained consent to participate following which the volunteer continued the phone call using the program’s standardised phone scripts as a guide. Call 1 was an introductory conversational call about health and well-being, providing health information on COVID-19, and assisting access to resources such as meals and utility support. Call 2 script enquired about older adult health including assessment of loneliness. UCLA asked in second call as loneliness sensitive topic so first call used to build trust. Call 3 assessed need for health and wellness services and other senior-related assistance programs available through the community service agency | NR | The community service agency paired older adults with student volunteers. The program aimed to identify the elderly's problems and establish a pathway for follow-up |
| Kramer 2022  [36] | NR | NR | Individual | Online eHealth service | Two embodied conversational agents represented as 2D humans in cartoon style talk (via text) with an older adult to motivate improved eating behaviour and decreased loneliness | NR | Consists of 5 modules, each applying different behavioural change techniques |

CBT=cognitive behavioural therapy; IPT=interpersonal psychotherapy; ToM=Theory of Mind

* Kall 2021 included an effective CBT intervention, though also included an ineffective IPT intervention

Φ Casanova 2021 (1)

# References

1. Casanova, G., Abbondanza, S., Rolandi, E., Vaccaro, R., Pettinato, L., Colombo, M., & Guaita, A. (2021). New Older Users’ Attitudes Toward Social Networking Sites and Loneliness: The Case of the Oldest-Old Residents in a Small Italian City. Social Media + Society, 7(4). <https://doi.org/10.1177/20563051211052905>
